# Paclitaxel-loaded elastic liposomes synthesised by microfluidics technique for enhance transdermal delivery

**DOI:** 10.1007/s13346-024-01672-0

**Published:** 2024-07-17

**Authors:** Eman Jaradat, Adam Meziane, Dimitrios A. Lamprou

**Affiliations:** 1 School of Pharmacy, Queen’s University Belfast, 97 Lisburn Road, Belfast, BT9 7BL UK; 2 Fluigent, Le Kremlin-Bicêtre, 94270 France

**Keywords:** Elastic liposomes, Microfluids, Emerging technologies, Chemotherapy, Paclitaxel

## Abstract

The inherent flexibility of elastic liposomes (EL) allows them to penetrate the small skin pores and reach the dermal region, making them an optimum candidate for topical drug delivery. Loading chemotherapy in ELs could improve chemotherapy’s topical delivery and localise its effect on skin carcinogenic tissues. Chemotherapy-loaded EL can overcome the limitations of conventional administration of chemotherapies and control the distribution to specific areas of the skin. In the current studies, Paclitaxel was utilised to develop Paclitaxel-loaded EL. As an alternative to the conventional manufacturing methods of EL, this study is one of the novel investigations utilising microfluidic systems to examine the potential to enhance and optimise the quality of Els by the microfluidics method. The primary aim was to achieve EL with a size of < 200 nm, high homogeneity, high encapsulation efficiency, and good stability. A phospholipid (DOPC) combined with neutral and anionic edge activators (Tween 80 and sodium taurocholate hydrate) at various lipid-to-edge activator ratios, was used for the manufacturing of the ELs. A preliminary study was performed to study the size, polydispersity (PDI), and stability to determine the optimum microfluidic parameters and lipid-to-edge activator for paclitaxel encapsulation. Furthermore, physiochemical characterisation was performed on the optimised Paclitaxel–loaded EL using a variety of methods, including Dynamic Light Scattering, Fourier Transform Infrared Spectroscopy, Atomic force microscopy, elasticity, encapsulation efficiency, and In vitro release. The results reveal the microfluidics’ significant impact in enhancing the EL characteristics of EL, especially small and controllable size, Low PDI, and high encapsulation efficiency. Moreover, the edge activator type and concentration highly affect the EL characteristics. The Tween 80 formulations with optimised concentration provide the most suitable size and higher encapsulation efficiency. The release profile of the formulations showed more immediate release from the EL with higher edge activator concentration and a higher % of the released dug from the Tween 80 formulations.

**Graphical Abstract:**

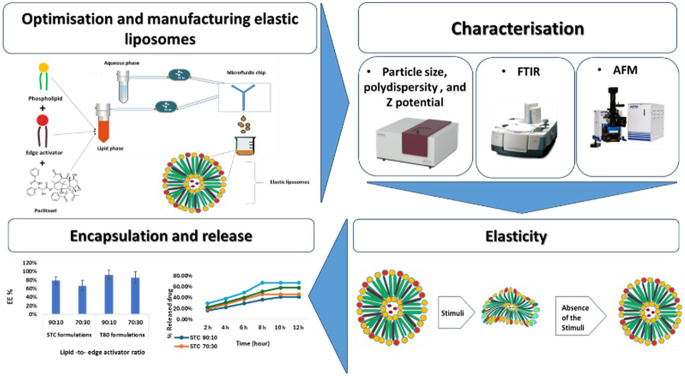

**Supplementary Information:**

The online version contains supplementary material available at 10.1007/s13346-024-01672-0.

## Introduction

**Fig. 1:**
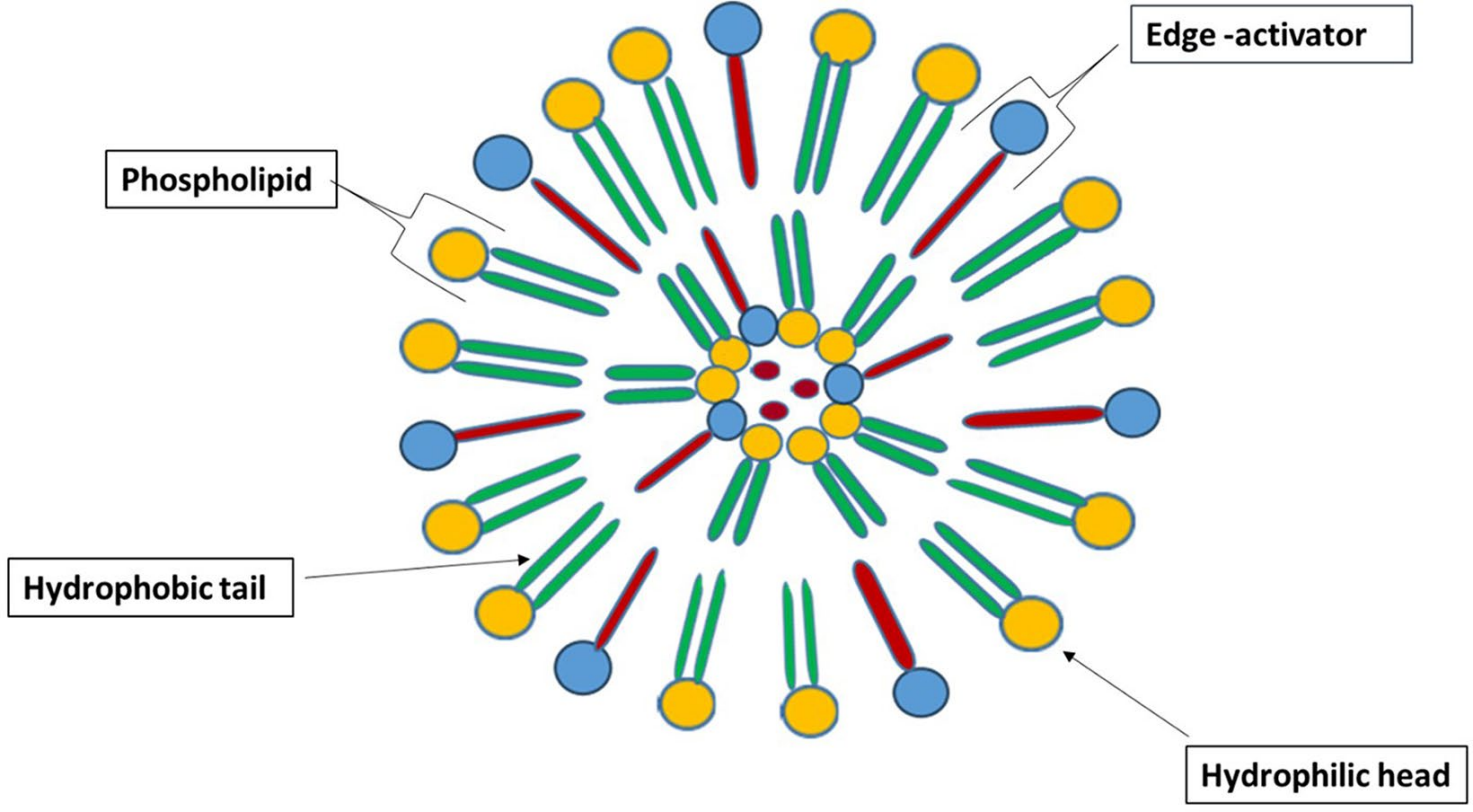
Schematic presentation of bilayer elastic liposomes displaying various components and structural morphology

Elastic liposomes (EL), an innovative version of conventional liposomal drug delivery systems (DDSs), have emerged as a promising area in pharmaceutical research and therapeutic applications. EL are characterised by their unique flexibility and adaptability, making them a versatile option for encapsulating various therapeutic agents, including chemotherapy [1]. Unlike conventional liposomes, elastic liposomes have a unique composition that includes edge activators or deformable lipid components (Fig. 1). This unique composition allows them to undergo reversible changes in shape when exposed to external stimuli. Edge activators are thought to be essential for giving liposomes their ultradeformability. Comprehending the impact of edge activators on the elasticity characteristics of EL is highly valuable for pharmaceutical applications in drug delivery. Choosing the optimal edge activators is crucial in the EL composition. Several researchers [2, 3] have demonstrated that edge activators are displaced to zones with elevated curvature/stress in a lipid bilayer as a result of mechanical stress. This displacement resulted in minimal energy expenditure when altering the forms and sizes of EL [4]. As a result, EL exhibits greater efficiency in delivering drugs than conventional liposomes.

The physicochemical properties of EL are influenced by various factors, including the type of edge-activator (e.g., ionic, non-ionic, and amphiphilic), the characteristics of the hydrocarbon chain that exist in lipids and edge activator (e.g., saturated, unsaturated, branching, and length), the transition temperature of the edge activator, the concentration, and the lipophilicity of the lipid, edge activator, and the active pharmaceutical ingredient (API) [5, 6]. The liposomes’ inherent elasticity imposes several advantages upon them as API carriers, such as the ability to facilitate efficient extravasation across biological barriers, the capacity to penetrate via tiny pores in the skin to reach the dermal region and targeting the delivery of APIs to specific area, which make them optimum for transdermal delivery [7]. In addition, ELs have numerous benefits, including protecting against degradation, achieving a high encapsulation percentage of lipophilic and hydrophilic molecules, providing a controlled release, and being cost-effective due to their lack of cholesterol [7, 8]. The efficiency of the EL as transdermal drug delivery (TDD) system has been reported by several previous studies [9–11]. The EL possess sufficient flexibility to undergo deformation and compression, enabling them to pass through the constricted intercellular gaps of the stratum corneum, which is the outermost layer of the skin. This capability facilitates the transportation of encapsulated drugs into deeper layers of the skin and potentially into systemic circulation. Various APIs have been encapsulated within the ELs of transdermal delivery, including antifungals, antibacterial, and chemotherapies [11–13]. The transdermal localized delivery of chemotherapeutics has been mainly investigated for the management of skin cancer.

Skin cancer is a life-threatening condition characterised by a high incidence of both cutaneous malignant melanoma and non-melanoma skin cancer, which has a global increase of over 600% since the 1940s [14, 15]. Skin cancer has become an increasingly serious health condition, with an annual incidence rate of 5–10% [14]. Different types of remedies have been used to treat skin cancer, including surgery, radiation therapy, and chemotherapy. Clinically, chemotherapy is one of the most frequently practised approaches to treat skin cancer [16]. However, chemotherapeutic drugs frequently have limitations such as low solubility and bioavailability, unfavourable pharmacokinetics, and non-selective biodistribution These limitations might complicate their clinical application and lead to undesired side effects [17]. Loading chemotherapy in ELs and utilising them as a transdermal DDS could be the optimum approach to localise the chemotherapy effect. The localised delivery of chemotherapies by EL might overcome the limitations of the conventional administration of chemotherapies. Also, the localised delivery of chemotherapy involves controlling the distribution of the drug to specific areas of the skin. Applying the chemotherapy-loaded EL directly to the tumour site leads to higher drug concentrations in the affected area while reducing exposure to healthy tissues. Different chemotherapies, such as cisplatin, 5-fluorouracil (5-FU), and paclitaxel, have been used for transdermal delivery to treat skin cancer.

Paclitaxel (PX), commonly referred to as Taxol, is a highly potent, efficacious, and commercially successful chemotherapy used for treating skin cancer [18]. The primary limitation of utilising PX arises from the drug’s limited solubility in aqueous solvents, below 0.1 mg/ml. Therefore, the loading of PX in special DDS is found to be one of the most effective and safest options to enhance the PX solubility. PX has been loaded in different types of DDSs for transdermal applications, especially lipid-based carriers, including micelles, conventional liposomes, and EL. However, ELs have been reported to be the optimum formulation for TDD systems. Multiple studies investigate the effectiveness of loading PX into EL and use them for transdermal formulations [13, 19]. However, several limitations have been reported for the EL formulations, such as large diameter, broad size distribution, and low encapsulation efficiency (EE). Researchers have established that the “pores” in the stratum corneum barrier are at least 10 times smaller than the average size of the general EL size reported until now, which usually exceeds 100 nm [20]. Also, the hair follicles and sweat ducts, the biggest pores on the skin, do not affect the penetration of liposomal transdermal drugs. The vesicle size of the transdermal formulations is one of the major parameters that will enhance the penetration of the formula onto the skin layers, which makes the nano DDSs superior for transdermal applications [16, 21]. The exceptional physical characteristics of nanoparticles (NP) make them ideal for use as transdermal carriers, owing to their capacity to adsorb and transport molecules and their high surface area to mass ratio [22]. Several studies have reported the efficacy of NP in facilitating transdermal delivery [16, 20]. Other studies have highlighted that EL with a diameter of 100 nm or larger showed substantially different patterns of tissue distribution and percutaneous absorption compared to those with a diameter of 200 nm or larger [23]. As the results reveals that EL with vesicle size less than 100 nm can deliver the drug to epidermis and dermas. In contrast to the EL, which has an average vesicle size of more than 200 nm and struggles to penetrate the brick-and-mortar construction of the stratum corneum [23]. The size of the EL is mainly affected by the manufacturing method. Usually, the ELs formulations manufactured using conventional methods such as, the thin film hydration method and vortexing-sonication method [13, 24]. The conventional manufacturing methods suffered from several drawbacks such as large and uncontrolled vesicles sizes, heterogeneous distribution of the vesicle size, consumption of organic solvents, and wasting large quantities of APIs and reagents. In this work, a novel investigation was conducted to examine the efficacy of the microfluidic (MF) system in optimising the EL formulations. MF is an innovative, sustainable, and adaptable technology that has been recently developed and utilised in several fields. A MF system can manipulate a small amount of fluid (ranging from 10^− 9^ to 10^− 18^ L) by utilising interconnected micrometre channels, microvalves, and micromixers. The primary characteristic of the MFs is their ability to provide a superlative fluid flow called laminar flow. This type of flow provides a parallel order flow at constant velocity without dispersing the layers, which consequently offers a high mixing quality and controls the size of the vesicles. In addition, it ensures consistent manufacturing quality throughout time, which enhances the homogeneity of the formulations and minimises batch-to-batch variability [22, 25]. The MF’s automated system provides high control over the manufacturing process parameters, including the total flow rate (TFR) and flow rate ratio (FFR). For the best of our knowledge, Since 2017, only one research study has used the MF system to manufacture ELs and encapsulate the AF-647 zoledronate drug for localised transdermal delivery [26]. The current research presented in this manuscript stands out as a first-of-its-kind examination of the MF system potency in manufacturing and optimising chemotherapy-loaded ELs for localised transdermal delivery.

In this work, a preliminary investigation was performed to explore the optimum TFR, FRR, and lipid-to-edge activator ratio. The work thoroughly focuses on enhancing the EL formulations for PX encapsulation by achieving a small size, low PDI, good stability, and high encapsulation efficiency.

## Materials and methods

### Materials

**Fig. 2:**
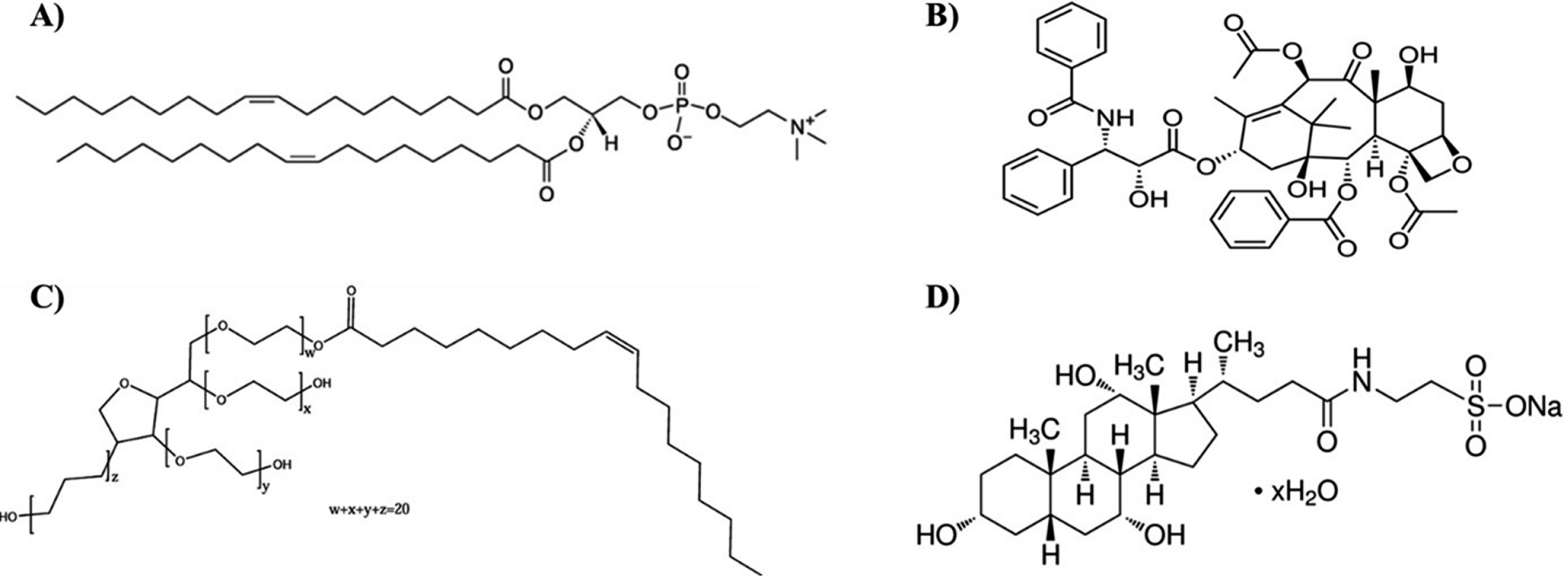
Chemical structures of: (**A**) 1,2-Dioleoyl-sn-glycero-3-phosphocholine (DOPC), (**B**) Paclitaxel, (**C**) Polysorbate 80 (T80), and (**D**) Sodium taurocholate hydrate (STC)

1,2-Dioleoyl-sn-glycero-3-phosphocholine (DOPC) and Paclitaxel were purchased form TCI (Portland, USA). Sodium taurocholate hydrate, phosphate-buffered saline (PBS, pH 7.4) tablets, Tween 80, ethanol (≥ 99.8%,) and acetonitrile (≥ 99.9%), were purchased from Sigma Aldrich (St. Louis, MO, USA). The chemical structure of the materials used can be seen in Fig. 2.

### Methods

#### Liposomal formulation preparations

**Fig. 3:**
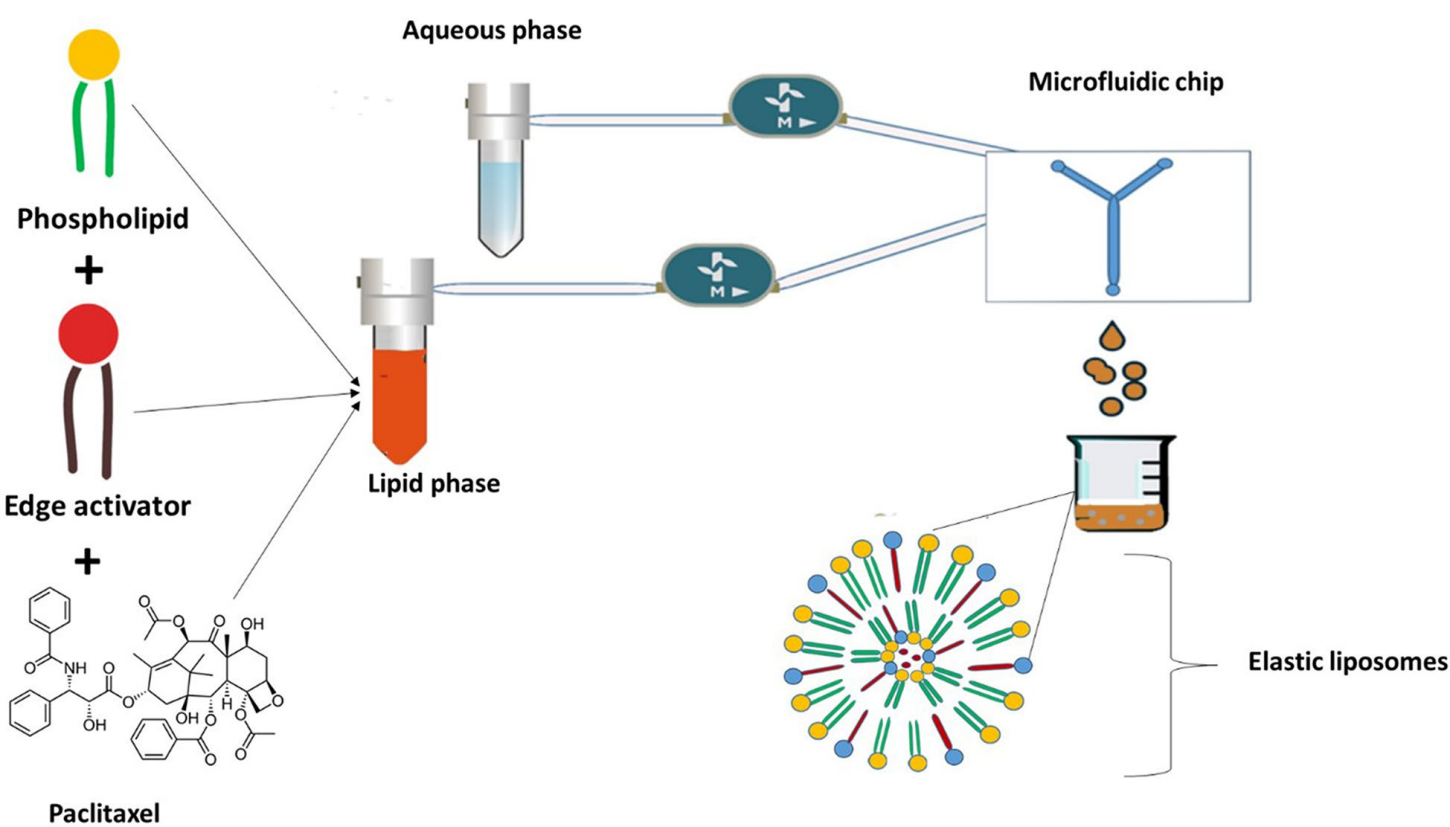
Graphic presentation for the MF system and the process of the EL manufacturing

The EL formulations were manufactured using FLUIGENT MFCS™ (Paris, France) automated hydrodynamic MF method (Fig. 3). A mixture of DOPC and different types of edge activators (T80 or STC ) are dissolved in ethanol (≥ 99.8% v/v) to prepare the lipid phase with a total lipid concentration of 5 mg/ml. The lipid concentration was determined based on the previously published lipid concentrations utilised in fabricating EL formulations [6, 7, 13]. Various DOPC-to-edge activator mass ratios were investigated to fabricate multiple empty EL formulations, including 90:10, 70:30, and 50:50 (as reported in Table 1 and table S1).

The pre-prepared lipid phase was sonicated to ensure the complete dissolving of the phospholipid and edge activator, then transferred to the MF reservoir. The aqueous phase of PBS (pH 7.4) was transferred to the other MF reservoir in parallel. Both aqueous and lipid phases are injected into a 3D-printed MF chip. The chip was fabricated using an Ultimaker 2 + FDM printer (Zaltbommel, Netherlands) and designed based on previous research conducted by our research group [27]. The aqueous and lipid phases are injected at different TFRs (1 and 2 ml/min) and FRRs (1:2, 1:3, and 1:4). The TFRs and FRRs ratios were selected to investigate the influence of varied ranges of MF parameters on the resultant ELs based on our previous study, which screened multiple TFRs ranging from low to highest applicable. The formulations were prepared in triplicates to allow for statistical analysis and reproducibility data.

**Table 1:** Lipid composition and lipid-to-edge activator ratios for the different formulations

Formulations	Edge- activator	Composition; DOPC: edge activator mass ratio	DOPC: edge activator molar ratio
Control	-	100% DOPC	-
F1	STC	90:10	0.86:0.14
F2	STC	70:30	0.64:0.36
F3	STC	50:50	0.42:0.58
F4	T80	90:10	0.94:0.06
F5	T80	70:30	0.79:0.21
F6	T80	50:50	0.64:0.36

The PX-loaded ELs were fabricated by adding PX to the lipid phase due to the PXT hydrophobic nature. The API was added and mixed with DOPC and the edge activator at a concentration of 0.1 mg/ml (1:10 drug to lipid mass ratio). This concentration was chosen based on previous literature findings, which indicate that a PXT concentration of 3–6% mol exhibits good solubility and remains stable for several weeks [28]. By calculating the number of moles of the used PXT con 0.1 mg/mL, the mol % was 6%. The PX-containing lipid phase was transferred to the lipid phase reservoir along with the PBS in the aqueous reservoir and injected into the MF chip. The phases are injected into the MF chip at the most suitable TFR and FRR, determined from the preliminary study of the empty ELs.

#### Characterization of liposomes

##### Vesicle sizing and ζ-potential

The vesicle size and PDI were determined by dynamic light scattering (DLS) with the Nanobrook Omni particle sizer (Brookhaven Instruments, Holtsville, NY, USA). A 20 µL aliquot of the liposomal formulation was diluted with 2 mL of PBS. All diluted samples were processed at 25 °C to measure the size and PDI. The identical approach was also employed to determine the ζ-potential. The measurement of each sample was repeated three times, utilising samples that were initially prepared in sets of three.

##### Stability study

The stability study was performed for the selected empty EL and the PX-loaded ELs. The diameter and polydispersity index (PDI) of the selected EL formulations were measured to examine the physical stability of the formulations. According to International Council for Harmonisation of Technical Requirements for Pharmaceuticals for Human Use (ICH) guideline Q1A for topical formulation stability, the selected formulation was kept in dark space for 4 weeks at 4 °C, 25 °C, and 37 °C to detect any aggregation [29]. Samples were withdrawn every week, and their size and PDI were observed by the DLS to verify their physical stability.

##### Fourier transform infrared spectroscopy

The empty and PX-loaded ELs were examined by FTIR analysis to investigate any potential interaction between the PX and the excipients and to analyse the effects of PX encapsulation on the stretching or bending of the chemical bonds. The analysis was conducted using an attenuated total reflection (ATR)-FTIR spectrometer (Thermo Fisher Scientific, Nicolet is 50 FTIR with built-in ATR, Massachusetts, U.S). The liposomal formulations were prepared by centrifugation at 14,800 rpm for 15 min. Subsequently, the remaining pellets were collected for examination. The liposome suspensions were analysed under an inert atmosphere covering a range of 4000–600 cm^–1^, with 64 scans conducted at a resolution of 4 cm^–1^ and an interval of 1 cm^–1^. Each sample underwent three tests, and all the samples were examined on day 0 to minimise the incidence of formulation degradation.

##### Atomic force microscopy

The AFM TT-2 AFM (AFMWorkshop, US) was employed to examine the empty and PX-loaded morphologies. A 20-µL volume of each sample is diluted with 2 mL of PBS water. Following this, 20 µl of the resultant solution was utilised to coat a cleaved mica measuring 1.5 cm × 1.5 cm (Agar Scientific Ltd., Essex, UK; G250–2 Mica sheets 1 in. × 1 in. × 0.006 in.). The solution is permitted to air dry for 30 min at room temperature. The samples were subjected to a thorough wash using 1 ml of PBS to remove any liposomes that were not attached to the mica surface. The solution was allowed to dry for a further 30 min prior of being examined by AFM. AFM images were acquired utilising Ohm-cm Antimony-doped Si probes with a resolution of 512 × 512 pixels, operating within a frequency range of 50–100 kHz, and a scan rate of 0.6 Hz. Three areas of the liposomal-coated mica were examined for every sample, and the most suitable images were used.

##### Elasticity

The elasticity of liposomes was assessed by following previous published method [6, 11]. The test was conducted for the optimised EL formulations and a conventional liposome formulation as a control. The control liposomes were prepared as discussed in 2.2.1; 100% DOPC was dissolved in ethanol and used as a lipid phase with a total 1 mg/ml concentration. The sample was then mixed with PBS as an aqueous phase at the same TFR of 1 ml/min and FRR of 1:4. The elasticity of the liposomes was measured by extruding the liposomal formulation through a polycarbonate microporous membrane with a pore size of 50 nm (Nuclepore, Whatman^®^) using Avanti^®^ polar lipids extruder (Alabaster, AL, USA) [6, 13]. The formulations extruded for 10 times at room temperature and the experiment was conducted three times. To determine the deformability, the average diameter was assessed by the DLS prior to and after the extrusion process. The membrane elasticity of the liposomes (E) was determined by Eq. 1. The elasticity of the liposomes was calculated using the following equation [30]:

1 $$\:E=J\left(\frac{rv}{rp}\right)2$$

Where, E = elasticity of liposome membrane; J = amount of suspension, which was extruded for 5 min; rv = liposome size (after extrusion); and rp = pore size of the membrane.

##### Encapsulation efficiency

According to the previously established method, a two-step centrifugation technique was conducted to measure the EE% of the EL. [7, 11]. In the initial stage, 1 ml of the liposomal suspension was centrifuged by (Thermo Fisher, HERAEUS PICO 21, Massachusetts, U.S) at 14,800 rpm for 30 min. After the initial centrifugation process, the resultant supernatant was withdrawn for further analysis. Following this, a volume of 1 ml of fresh PBS was introduced into the precipitate, and a subsequent round of centrifugation was performed to extract any drug that might have been adsorbed physically onto the liposomal surface. Three repeats were conducted for every formulation. The supernatants generated from these centrifugation processes were analysed using Ultraviolet High-Performance Liquid Chromatography (UV-HPLC). A C18 column (250 mm × 4.6 mm) from Thermo Fisher Scientific( Massachusetts, U.S) was used to analyse the free drug, with detection at 227 nm [31]. The samples were analysed using isocratic elution with a mobile phase consisting of a 50:50 gradient of acetonitrile and water [32, 33]. The injected sample had a volume of 50 µL, and the overall flow rate was 1 ml/min. The EE% of the PX-loaded EL was determined using Eq. 2.

2 $${E}=\frac{\begin{array}{l}{{Total}\:{amount}\:{of}\:{the}\:{add}\:{drug}\:\left({mg}\right)}\\{-{free}\:{drug}\:{amount}\left({mg}\right)}\end{array}}{{Total}\:{amount}\:{of}\:{the}\:{drug}\:\left({mg}\right)}\:\:\:\times\:100{\%}\:$$

##### In vitro release

The in vitro release profile of PX from the EL was investigated using the dialysis tubing method. This investigation utilised dialysis bags containing a cellulose membrane with an average flat width of 10 mm (0.4 in.) and a molecular weight cutoff (MWCO) of 14,000, obtained from Sigma Aldrich. Prior to analysis, the dialysis bags were sterilised by boiling in deionised (DI) water and subsequently rinsing with DI water. The samples were prepared by centrifuging 1 ml of each EL formulation at 14,800 rpm for 30 min. The resultant supernatant was eliminated, and the participating liposomal pellets were hydrated with PBS water and then placed into the dialysis bags. The dialysis bags were submerged in a release medium composed of 6 ml of PBS (7.4 pH). Subsequently, samples were moved to a 37 °C incubator to commence the release study. The sample for analysis (1 mL) was withdrawn at pre-determined intervals (2, 4, 6, 8, 10, and 12 h) and was replaced with fresh buffer to maintain skin conditions [24].

##### Statistical analysis

All experiments were conducted three times, and the mean and standard deviation were calculated. One-way ANOVA tests have been performed for the stability study of both 70:30 and 90:10 ratios, and for the diameters, PDI, and ζ-potential of the empty and PX-loaded of STC and T80 formulations.

## Results and discussion

### Preliminary study to determine the TFR, FRR, and lipid-to-edge activator ratio

This study aimed to assess the efficiency of the MF system in manufacturing optimal ELs for dermal drug delivery. The leading goal in optimising EL formulations for dermal drug delivery is to achieve ELs with a size < 200 nm, PDI below 0.25, and exhibit good stability and homogeneity. The optimisation involved the manufacturing method parameters, including TFR and FRR, and the lipid-to-edge activator ratio. The manufacturing method was optimised by investigating the MF parameters, including the TFR and FRR, to determine the most suitable parameters. To optimise the nanosystem of ELs, DOPC was incorporated with two different edge activators (T80, STC) at various phospholipid-to-edge activator ratios. DOPC was selected due to its high elastic compliance; lipids with unsaturated alkyl chains have a less packed bilayer and hence free spaces, making them ideal for incorporating edge activators [34]. Both of the edge activators were used with the DOPC to study the impact of the edge activator type on the ELs characteristics; T80 is used as a non-ionic edge activator, and STC is used as an anionic edge activator. The lipid-to-edge activator ratio should be optimised to ensure high stability and improved elasticity, hence facilitating skin permeability and long-term stability. Therefore, the most optimal phospholipid-to-edge activator ratios were determined to be utilised for PX encapsulation.

#### The effect of total flow rate and flow rate ratio

**Fig. 4:**
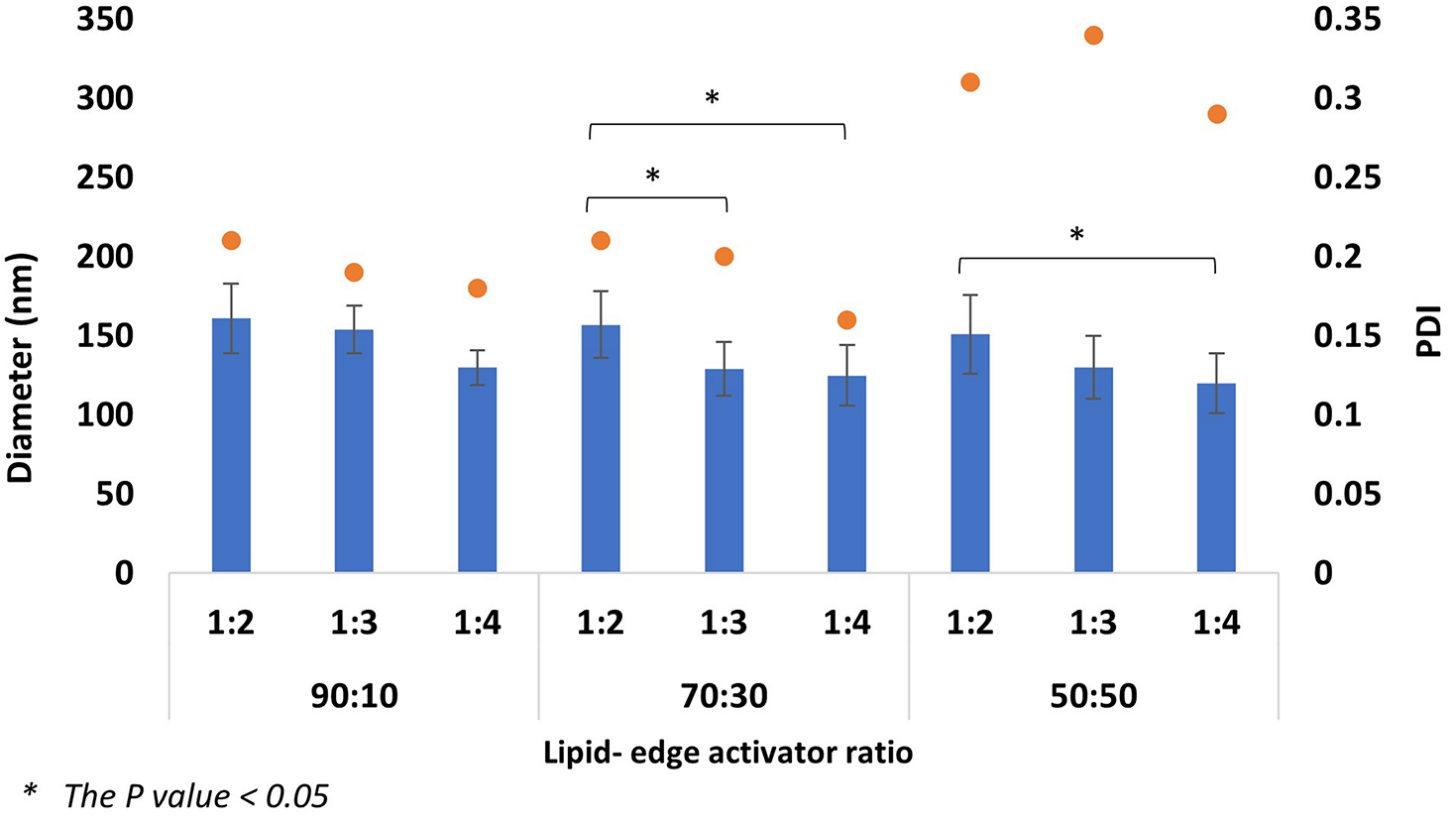
Average diameter of STC elastic liposomes diameter at TFR 1 ml/min and FRR 1:2,1;3, and 1:4

**Fig. 5:**
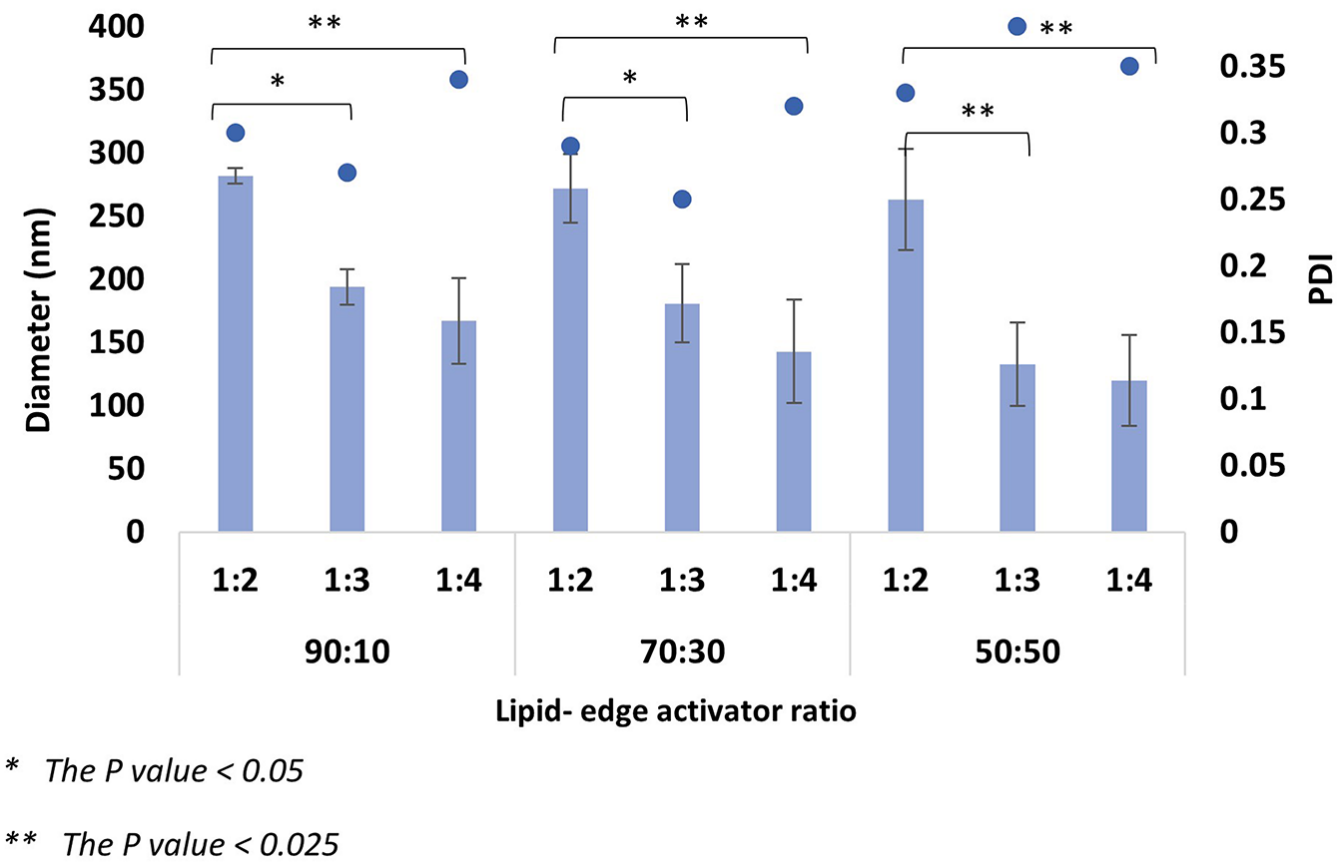
Average diameter of STC EL diameter at TFR 2 ml/min and FRR 1:2,1;3, and 1:4

**Fig. 6:**
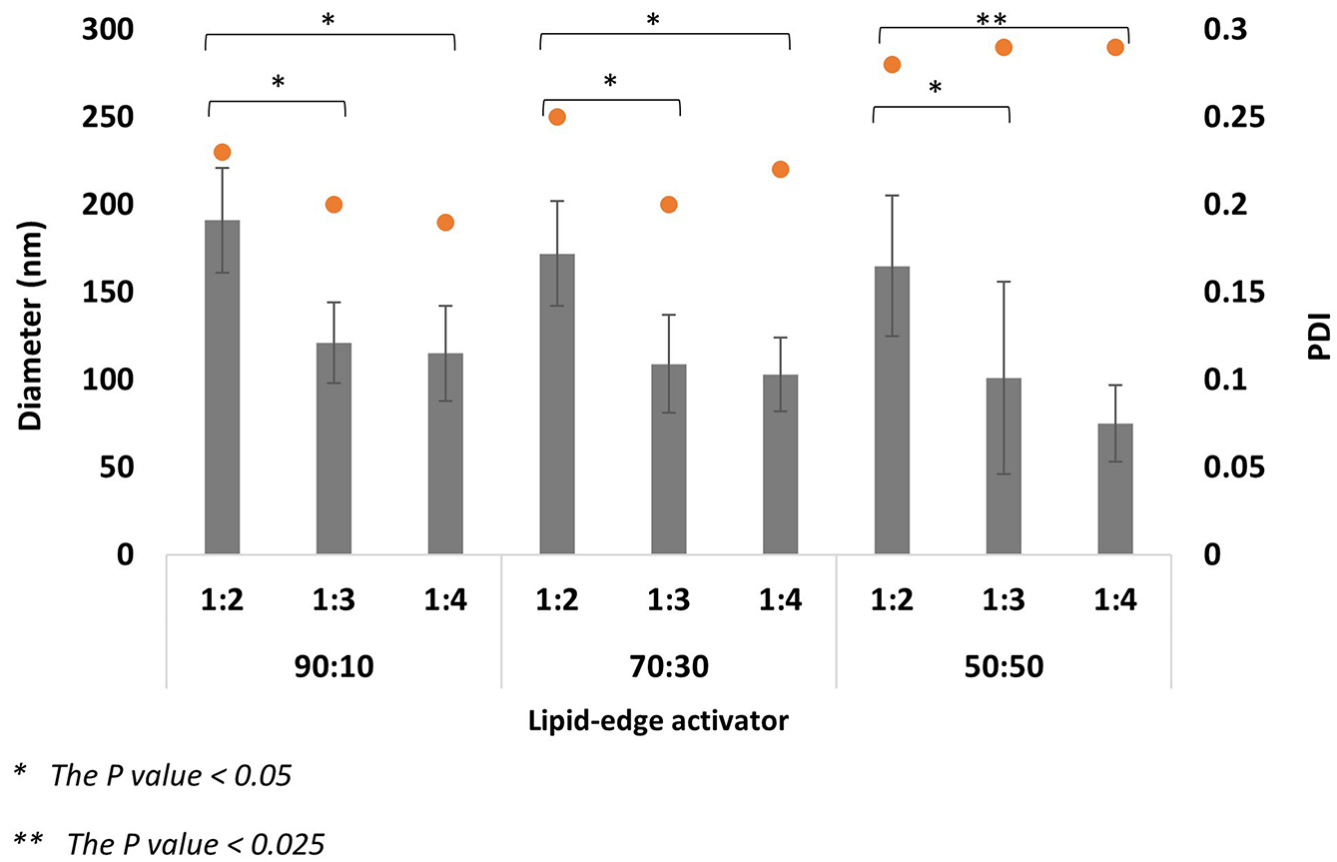
Average diameter of Tween 80 elastic liposomes diameter at TFR 1 ml/min and FRR 1:2,1;3, and 1:4

**Fig. 7:**
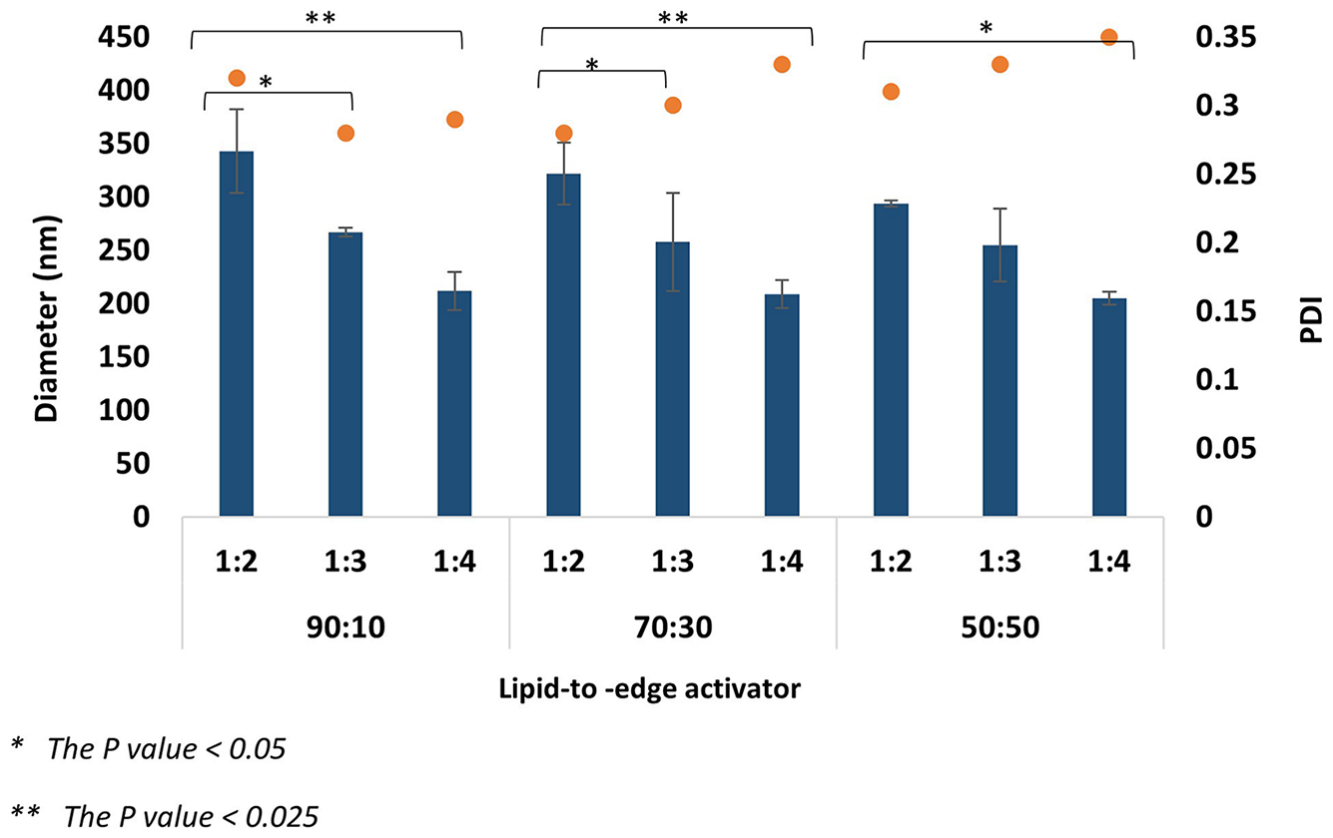
Average diameter of Tween 80 elastic liposomes diameter at TFR 2 ml/min and FRR 1:2,1;3, and 1:4

The MF system was used for ELs manufacturing through different TFRs (1 and 2 ml/min) and FRRs (1:2, 1:3, and 1:4). The variation of the MF parameters, especially TFR and FRR, highly affects the EL diameter and PDI. Changing the TFR from 1 to 2 ml/min impacted the formulation’s size and homogeneity (Figs. 4, 5, 6 and 7). By comparing both TFR, the most suitable formulations were produced at TFR 1 ml/min; changing TFR from 1 to 2 ml/ min increased the ELs’ diameter and affected the constancy of both T80 and STC formulations (Figs. 4, 5, 6 and 7). At TFR 1 ml/min, the vesicle size of the various lipid-to-edge activator ratios, including 90:10, 70:10, and 50:50 of ELs, was smaller, more homogeneous, and reproducible with lower standard deviation (SD). The average diameter of the different ratios of STC ELs at TFR 1 ml/min was 140 ± 16 nm compared to the average diameter at TFR 2 ml/min which was 195 ± 62 nm. The same trend was noticed with T80 formulations; the average diameter of the different ratios at TFR 1 was 128 ± 38 nm and increased to 262 ± 49 nm when the TFR increased to 2 ml/min. Also, the results showed that by increasing the TFR at a specific FRR, an increase in the EL diameter was noticed. For example, the 90:10 of STC ELs at TFR 1 ml/min and FRR 1:2 was 161 ± 22 nm, and by increasing the TFR to 2 ml/min at the same FRR, the diameter increased to 282 ± 6 nm. The same enlargement of the EL size when the TFR increased at specific FRR was noticed in T80 formulations (Figs. 5 and 6).

The linkage between the TFR and ELs diameter can be clarified by comprehending the liposome’s creation process via lipid and aqueous solvent mixing. Once the lipid and aqueous solvents are exchanged and mixed, the bilayer fragments (BFs) are formed firstly at the water–organic solvent interface [35]. When the polarity of the mixture starts to increase, the BFS fragments start to assemble as a liposome. Here, utilising MF, by increasing the TFR to 2 ml/min, the strength of the secondary flow increases, which might result in an increased propensity of BFS to cluster and aggregate as supramolecular lipid aggregates [36].

These findings are consistent with our previous work, which investigated the impact of TFR on conventional liposome diameter [33, 37]. Other studies reported the same trend of the linear relationship between the liposome’s diameter and TFR [36]. The impact of the TFR on the PDI was more consequential and significant; by increasing the TFR from 1 to 2 ml/min, the average PDI of the STC formulations increased from 0.23 to 0.34, respectively. The same trend was observed in T80 formulations; the PDI average of the T80 at TFR 1 was 0.24 ± 0.04, and by increasing the TFR to 2 ml/min, the PDI increased to 0.31 ± 0.02. Moreover, by studying the formulation ratios individually to investigate the effect of increasing the TFR on the PDI at the same edge activator to lipid ratio, the PDIs of 90:10, 70:30, and 50:50 ratios of STC ELs at TFR 1 m/min were 0.19, 0.2, and 0.31. By increasing the TFR to 2 ml/min, the PDI of ratios increased to 0.3, 0.29, and 0.35, respectively. Similar findings were noticed with T80 ELs; the PDIs of the 90:10, 70:30, and 50:50 formulations at TFR 1 were 0.21 ± 0.02, 0.22 ± 0.03, and 0.28 ± 0.01, and after increasing the TFR to 2 ml/min the PDI averages increased to 0.29 ± 0.02, 0.30 ± 0.03, 0.33 ± 0.02, respectively. The increase in the PDI values when the TFR increased was significant (*P* = 0.006), which indicates the production of aggregates or non-homogenous EL at TFR 2 ml/min. Moreover, the relatively high SD values of the different formulations at TFR 2 ml/min might support these findings and show unreproducible formulations.

The FRR was another major factor impacting the ELs diameter and PDI with an inverse relationship; increasing the FRR from 1:2 to 1:4 at specific TFR provides ELs with smaller diameters, which mimics the trend of several previous studies [26, 36, 38, 39]. By comparing the impact of the TFR and FRR, the FRR was reported to significantly impact the EL’s diameter (*P* = 0.024). The decline in ELs diameter with increasing the FRR was observed at all the different ratios of STC and T80 ELs formulations, including 90:30, 70:30, and 50:50 (Figs. 4, 5, 6 and 7). For example, by observing the 90:10 T80 Els diameter trend at TFR 1 ml/min, the largest diameter obtained at FRR 1:2 was 191 ± 30 nm, and by increasing the FRR to 1:3 and 1:4, the Els diameter decreased to 121 ± 23 and 115 ± 27 nm, respectively. Likewise, the smallest STC ELs diameters were produced at TFR 1 ml/min and 1:4 FRR including the different ratios; 130 ± 18 nm for 90:10, 125 ± 19 nm for 70:30, and 120 ± 29 nm for 50:50. For T80, the same trend reported; as the smallest diameters of the ELs obtained at TFR 1 ml/min and 1:4 FRR with 115 ± 27 nm for 90:10, 103 ± 21 nm for 70:30, and 75 ± 22 nm for 50:50. The reduction in the ELs’ diameter might occur due to the decrease of the final lipid solvent concentration at high FRR; reducing the final lipid solvent concentration has been reported to inhibit particle fusion and merging and limit the occurrence of the Ostwald ripening phenomenon [40, 41]. The impact of the FRR on the PDI was minor and varied. By studying the trend of the PDI values in both STC and T80 formulations with 90:10 and 70:30 Els, a decrease in the PDI values has been noticed after increasing the FRR from 1:2 to 1:4 (Figs. 3 and 5), especially at TFR 1 ml/min, which resemble the trend of other previous studies [26, 42]. However, by changing the lipid-to-edge activator ratio to 50:50 at the same TFR, no linear trend was noticed between the PDI and the FRR.

The physical stability of the various lipid ratios at the different FRRs, including 1:2, 1:3, and 1:4, were measured over four weeks to ensure low aggregation incidence and appropriate liposomal formulation homogeneity (Figures S3–S13). Generally, the Vesicle size and SD increased at 37 °C compared to 4 °C and room temperature, which remains relatively constant. The results represent an impact of the FRR on the stability of the formulation; the results indicate that the formulation produced at FRR 1:4 exhibits the highest stability among the tested formulations. This finding validates the vesicle size results and confirms the superiority of the 1:4 FRR.

#### The effect of lipid-to- edge activator ratio

The ELs compose unsaturated phospholipids as the main constituent and edge activator in a specific ratio [43]. The DOPC phospholipid was chosen due to the unsaturated acyl chain and the low transition temperature (Tm), which provide higher membrane flexibility and elasticity [20]. The DOPC-to-edge activator ratio should be optimal due to the significant and vital effect on the ELs’ elasticity and stability. Different DOPC-to-edge activator mass ratios have been investigated, including 90:10, 70:30, and 50:50. The rationale behind the selected ratios was to investigate the effect of a wide range of edge activator concentrations, starting from a low concentration of 10% to a high concentration of 50%. The results represent a significant effect of the variation in the DOPC-to-edge activator ratios, either T80 or STC, on the liposome diameter and PDI. The 90:10 and 70:30 ratios produced the most suitable EL formulations compared to the 50:50 ratio. For example, the average diameter of the different formulations of STC 90:30 formulation at TFR 1 ml/min was 148 ± 16 nm, 137 ± 17 nm for 70:30, and 133 ± 16 for 50:50. The same trend of decreasing the liposome diameter after increasing the T80 ratio was reported. Several studies reported the same trend of reducing the EL diameter after increasing the edge activator concentration [24, 44, 45]. The reduction in the EL’s size when the sedge activator ratio increased might be due to micelles’ formation [46]. The PDI results support this hypothesis, as increasing the edge activator ratio for both T80 and STC showed a significant increase in the PDI values and the heterogeneity of the formulations (Figs. 4 and 6). A linear relationship was noticed between the PDI and the edge activator concentration, either T80 or STC; increasing the edge activator ratio from 90:10 to 50:50 results in a growth of the PDI values. For example, the average PDI of the STC formulations at 90:10 was 0.19, then increased to 0.2 and 0.31 for 70:30 and 50:50, respectively. The same linear trend was noticed with T80 formulations, as the average PDI values were 0.20, 0.22, and 0.28 for 90:10, 70:30, and 50:50, respectively. Several previous studies have reported that increasing the edge activator concentration to more than 15% can influence the formation of micelles and increase the incidence of aggregation [24, 44, 46]. The formation of micelles can affect the homogeneity and stability of the formulations and reduce the ELs’ EE due to the micelles’ limited EE compared to the liposomes. Overall, the PDI of formulation is as important as the EL diameter in optimising the EL nanosystems due to its effect on the EE, formulation efficacy, and appearance [47]. The 90:10 and 70:30 ratios were determined as the most suitable ratios due to the optimum diameter (< 200 nm) and PDI (< 0.25), which are both critical for achieving superior DDs.

Moreover, the ζ-potentials of STC and T80 edge activator formulations were evaluated to investigate the empty ELs electrostatic charge (Figures S1 and S2). The utilised edge activators are varied in type; STC is an anionic edge activator, and the T80 is a natural edge activator. The result demonstrated a -13.7 MV average ζ-potential for the various ratios of the STC formulations and − 6 MV for the T80 formulations. For STC formulations, the increase of the edge activator ratio from 90:10 to 50:50 results in driving the electrostatic charge of the ELs to be more anionic. This can be explained by boosting the negative charge intensity by increasing the anionic edge activator concentration. For T80 formulations, the variation in the edge activator ratios does not affect the total electrostatic charge of the ELs. Although using a neutral edge activator, the liposome’s electrostatic charge was slightly anionic due to the positioning of the negative phosphate group towards the liposome surface, as opposed to the choline group. The theory argues that the choline group, which is slightly hydrophobic due to the presence of methyl groups at the nitrogen end, is orientated towards the interphase of liposomes in order to prevent contact with the aqueous phase [48, 49]. The ζ-potential of liposomes has a significant role in determining their properties, particularly in terms of formulation stability and indicating the pharmacological interactions of the vesicles [50]. The more anionic ζ-potential confers a benefit by enhancing the stability of the liposomes since it leads to an increase in repulsion forces among the liposomes, inhibiting aggregation [51]. Furthermore, liposome compositions with a negative charge significantly enhanced the permeation of EL in transdermal delivery. The skin possesses a minor negative charge [52]. Hence, the negative ζ-potential may enhance the penetration of EL through the skin and boost their accumulation in the superficial layers due to the virtue of the electrostatic repulsion between the skin surface and liposomes [10, 53]. On the other hand, for skin DDSs, the electrostatic charge is preferred to be neutral or slightly anionic; the pH of the skin is slightly acidic, and the application of highly cationic or anionic formulation might result in serious irritation to the skin. Furthermore, additional research has reported that the slightly negative vesicles result in improved adsorption onto and fusion with the stratum corneum and consequent enhancement in transdermal drug delivery [54].

To ensure the adequate stability of both selected ratios as empty EL carriers, a 4-week stability study was performed for the 90:10 and 70:30 ratios at 4 °C, 25 °C and 37 °C (Figures S3 to S13). The first two temperatures were examined as a storage condition, whereas the 37 °C was chosen to mimic the physiological state of the body temperature. The stability studies results showed a significant vesicle size and SD increase at 37 °C compared to 4 °C and room temperature, which stayed relatively constant. The increase in the EL size at 37 °C occurred due to the loose packing of the lipid bilayer as the temperature increased, which led to a rise in the overall volume of the EL [55]. Both ratios generally showed good stability as the vesicle size was comparable, and no significant difference was noticed during the 4 weeks, especially at FRR 1:4. The good stability of the EL at both ratios indicates their eligibility to be used as a drug carrier.

The most optimum parameters have been determined based on the parliamentary study of the TFR, FRR, and lipid-to-edge activator ratio. For the manufacturing method parameters, the TFR 1 ml/min produced EL with a smaller size and high homogeneity, making it more suitable than TFR 2 ml/min. At TFR 1 ml/min and among the various studied FRRs, the FRR 1:4 produced the smallest EL diameter with suitable PDI and high stability. Several DOPC to edge activator ratios were investigated for STC and T80, including 90:10, 70:30, and 50:50. The variation in the used edge activator did not show any discrepancy in the result; all the parameters impact and the trend of the result was similar for STC and T80 were similar. The 90:10 and 70:30 ratios were the optimum due to the high homogeneity and stability of the resultant formulations. The optimal EL formulations were obtained using the 90:10 and 70:30 DOPC to edge activator ratios at a TFR of 1 ml/min and a FRR of 1:4. These formulations are appropriate for PX encapsulation due to their optimal size, PDI, and excellent stability.

### Characterisation of PX-loaded EL

#### Vesicle size, PDI, and ζ-potential

The small Diameter of the EL is a critical factor for enhancing the effectiveness of the skin DDSs; the small diameter of the ELs facilitates their penetration into the skin to reach deep layers, offering a larger surface area for drug interaction with the skin, which enhances API absorption through the stratum corneum, and provide smoother texture for formulation which reduce any irritation and improve the patient’s compliance. Several studies reported that vesicles with a diameter of less than 300 nm possess the capability to partially deliver their contents into the underlying layers of the skin [56–58]. From the preliminary study, four formulations have been determined for PX encapsulation due to their optimum diameter, PDI, and stability. PX is a highly prevalent antineoplastic medication employed for the treatment of several types of skin cancers, including squamous cell carcinoma [59], melanoma [60], and basal cell carcinoma [61]. The clinical application of PX is still limited due to the low water solubility, insufficient release amount, and high molecular weight (853 Da) that limit its penetration to the skin [60, 62, 63]. The encapsulation of PX in nano EL might overcome these limitations and improve PX skin penetration.

**Fig. 8:**
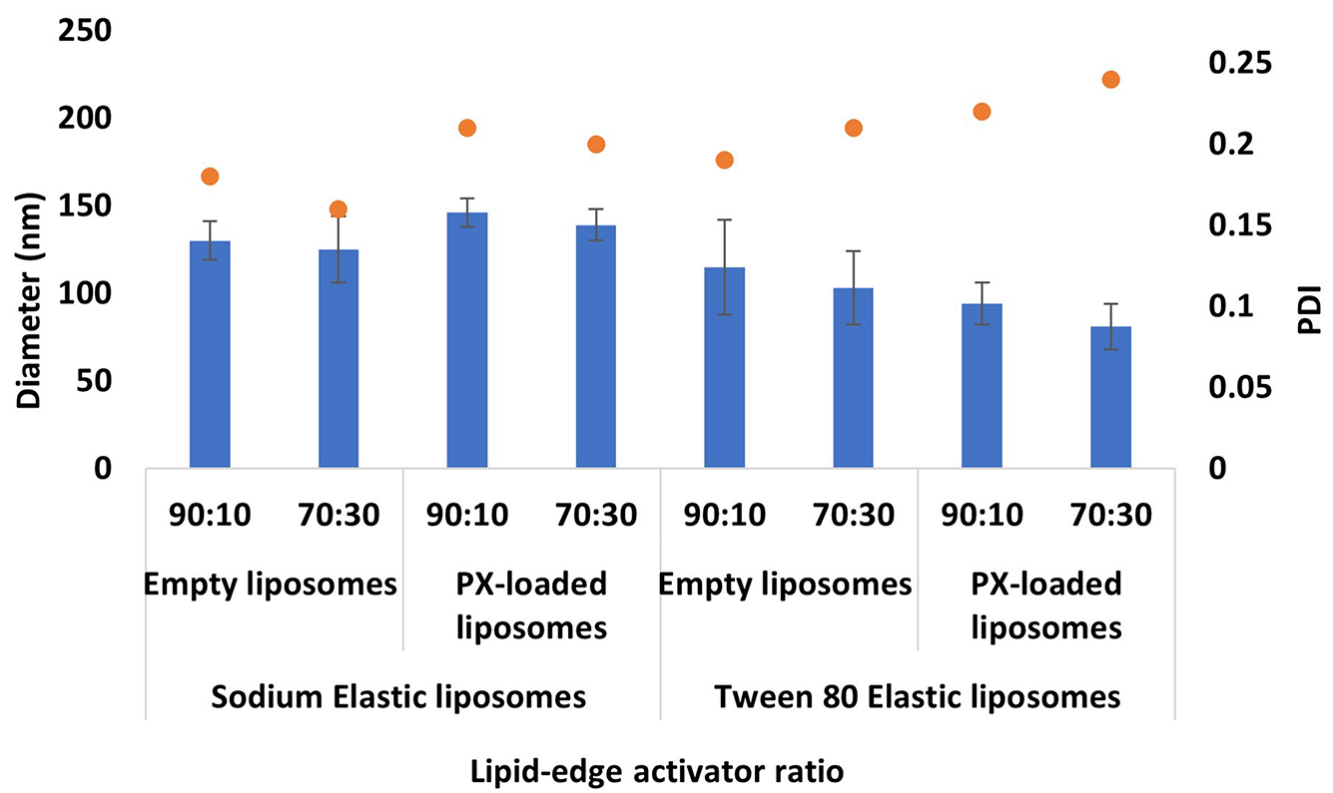
Average diameter of empty and PX- loaded EL diameter at TFR 1 ml/min and FRR 1:4

The 90:10 and 70:30 DOPC to edge activator ratio at TFR 1 ml/min and 1:4 FRR produced the most suitable formulations for PX encapsulation due to the small diameter (< 200 nm), low PDI (< 0.25), and high stability. The determined parameters have been utilised for both edge activators, including STC and T80, which provide four different formulations of PX-loaded EL. The free drug EL and PX-loaded EL measurements were comparable; no significant difference was reported between the diameters. By comparing the different edge activators, the diameter of the T80 ELs was relatively smaller than STC ELs even before the PX encapsulation (Figs. 4 and 6). This was attributed to the longer carbon chain length of the T80, which is associated with increasing the hydrophobicity of the T80 and improving the solubility of the T80 within the lipid bilayer. Several studies highlighted the impact of the carbon chain length and the edge activator’s hydrophobicity on reducing the liposome’s diameter [46, 64, 65]. For example, Duangjit et al. studied the impact of utilising various edge activators with different carbon chain lengths on the meloxicam-loaded liposome diameter [64]. The results reported that the edge activator with a longer carbon chain length represents higher hydrophobicity and enhances the solubility of the edge activator in the bilayer, which decreases the overall diameter of the liposomes. Following the PX encapsulation, the STC EL diameter increased, and the T80 EL diameter decreased (Fig. 8). For example, the diameter of the 90:10 T80 formulation was 115 ± 27 nm and decreased to 94 ± 12 nm after PX encapsulation, and the diameter of the 90:10 STC formulation was 130 ± 11 nm and increased to 146 ± 8 nm after PX loading. The disparity in diameter following PX encapsulation may primarily arise from the variance in edge activator and PX structure. The T80 molecule consists of a hydrophilic part characterised by a long carbon chain in contrast to the STC structure, which has a steroidal structure like the PX structure (Fig. 2). The hydrophobic nature of PX and the presence of a longer carbon chain in T80 enhance the PX solubility and drive a tight packing of PX inside the bilayer due to the robust van der Waals construction, which reduces the average diameter of the EL [46, 66].

The PDI values remained relatively comparable after PX encapsulation, since no statistical difference was noticed. A minor elevation in the PDI values was noticed after incorporating PX into the ELs. The elevation in the PDI might be due to the packing of the PX within the bilayer; the incorporation into the bilayer may introduce a steric hindrance factor during the formation of the bilayer [67]. This factor can be one of the causative factors for the PDI elevation upon encapsulation. However, all the formulations` PDI values remained < 0.25, which indicates homogenous formulations. The promising results of the PDI can be mainly attributed to the superior mixing quality of the MF system. Analogising liposomes produced using conventional methods revealed that the PDI values exhibited a twofold increase following PX encapsulation [68].

**Fig. 9:**
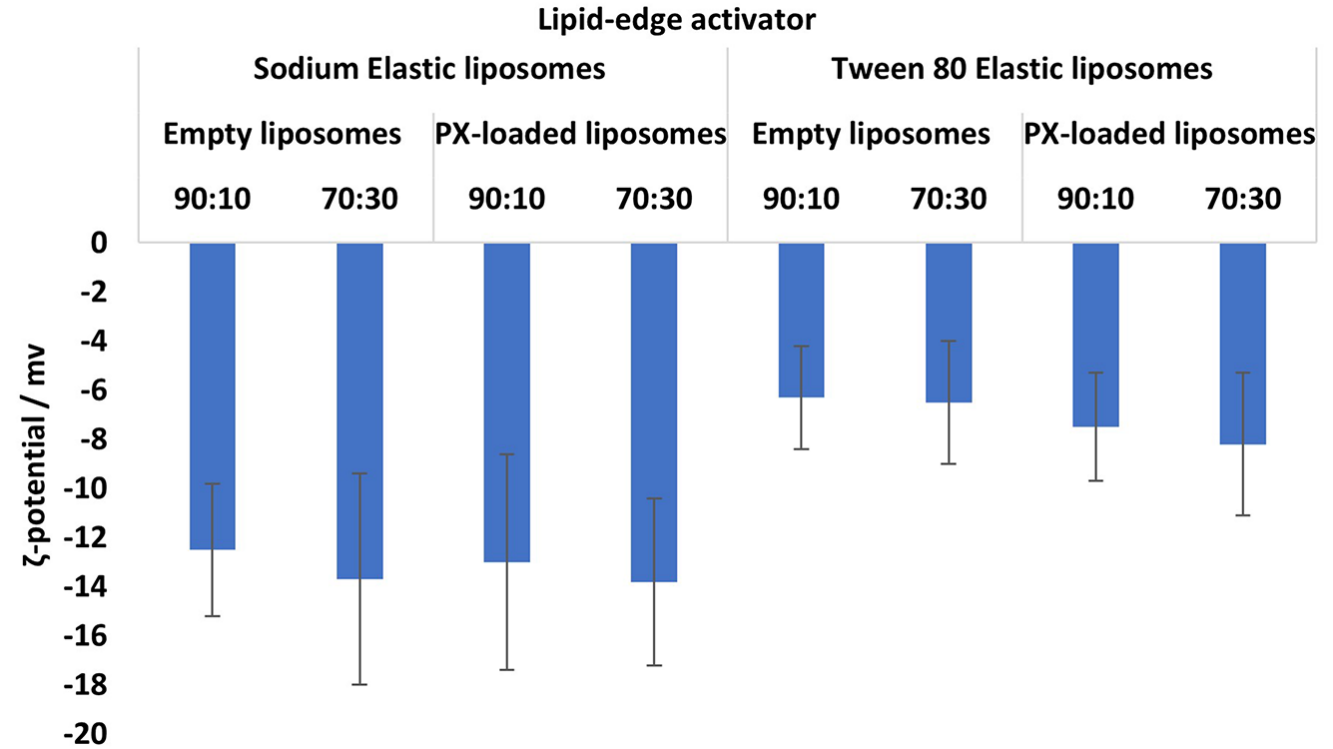
Average ζ-potential of STC and T80 Paclitaxel- loaded elastic liposomes

The ζ-potential of the formulations has been measured before and after PX encapsulation. The results represent comparable charges, as no significant statistical difference was reported between the empty and the loaded ELs. The ζ-potential of the formulations becomes barely more anionic for both T80 and STC formulations (Fig. 9). The variation in the edge activator type is the main reason for the disparity in the formulation charge; the STC edge activator with anionic charge provided formulation with an average ζ-potential of -13.4, and the T80 with neutral charge provided formulation with an average ζ-potential of -7.85. The ζ-potential has a crucial role in determining the features of liposomes, particularly their stability and the pharmacological interactions of the molecules [49, 50, 69]. Determining the more appropriate formulation, anionic or neutral, depends mainly on the targeted area and the deposition location [69]. However, for formulation development, the enhancement in the anionic charge mainly improves the stability of the formulation and lowers the incidence of any aggregates [51, 69]. Stability studies have been performed on the various loaded EL formulations to investigate the impact of the edge activator type on stability.

#### Stability study of loaded elastic liposomes

The physical stability of the loaded ELs was performed over four weeks to investigate the vesicle size and dispersity of the formulation (Figures S14 and S15). Previously, the physical stability of the empty EL was studied to ensure its stability as a carrier. Here, the physical stability of the PX-loaded EL was performed to ensure the stability of the ELs as a DDS. An increase in the vesicle size of the loaded STC and T80 ELs was seen as compared to the empty formulations at a TFR of 1 ml/min and a FRR of 1:4. Despite the increase in the vesicle size of the EL after PX encapsulation, the stability of the formulation improved at different temperatures, particularly at 37 °C. As an illustration, the mean vesicle size for the empty 90:10 STC EL at a temperature of 37 °C was 185 ± 22 nm, whereas for the loaded formulation under the same conditions, the diameter was 173 ± 18 nm. The vesicle size difference between day 0 and after incubation at 37 °C was smaller in the loaded liposomes for both edge activators. The continued stability of PX-loaded ELs vesicle size at 37 °C may be attributed to the tight packing of PX molecules inside the liposomes bilayer and the generated interactions (ex, van der Waals) between the drug and the hydrocarbon chains [66]. Upon further investigation of each edge activator separately, the STC formulations exhibited high stability before and after PX encapsulation. This can be attributed to the anionic charge of the edge activator, which generates repulsive forces and inhibits the aggregation of the liposomes. However, the T80 formulations showed a significant stability enhancement after PX encapsulation. The deviation in vesicle size between day 0 and during the four weeks was measured, and it was found that the EL diameters remained consistent and stable across various temperatures, particularly at 37 °C. Also, the average PDI was determined to be 0.21. The variation in hydrocarbon chain length is probably the cause of this phenomenon. As explained in Sect. “Vesicle size, PDI, and ζ-potential”, the T80 has a longer hydrocarbon chain, which promotes the solubility of PX molecules in the bilayer. Additionally, it enhances the lateral packing of molecules within the membrane due to the stronger van der Waals [46, 66].

#### Spectroscopic evaluation

FTIR analysis was performed for both empty and PX-loaded STC and T80 Els (Figure S16–S19). FTIR can be used to detect minor modifications in the structure and function of lipid assemblies. It achieves this by examining alterations in the frequency or bandwidth of several vibration modes corresponding to lipid molecules’ acyl chains, interfacial area, and head group region. The empty formulation was used as a control to investigate the changes in the vibration of the chemical bonds and to determine if any newly generated bonds emerged after loading the PX. The observed peaks exhibit distinct functional groups depending on the utilised edge activator. Still, multiple functional groups were observed in all the samples, including the O–H bond, which is present at the wavelength range of 3218–3349 cm^–1^, indicating the presence of a primary alcohol group. The O–H peak may be attributed to residual ethanol employed in the formulation manufacturing. Also, the C–H bond peaks were observed at 2850–2958 cm^–1^, indicating stretching in the symmetric region of the alkane chain. The PO4 − group is a primary functional group of the DOPC lipid. The phosphate peaks identified in the region of 1270 cm^− 1^ indicate symmetric stretching vibrations [70, 71]. The resulting peaks of the empty formulations showed a variation in the wavelength of spectra due to the different functional groups in the STC and T80 structures. For example, in the empty STC formulation spectra, the S = O bond of the sulfur dioxide showed in two sharp Peaks at 1085 and 1044 cm^− 1^. The oxygen-containing absorptions appeared within a crowded and highly overlapped region of the spectrum, mainly between 1350 and 950 cm^− 1^ [71, 72]. Here, two sharp peaks at the 1085 and 1044 cm^− 1^ correlated to the asymmetric stretching of the S = O bond. Another peak corresponding to NH bending of the amide functional group has been noticed at 1643 cm^− 1^. A comparison between the resulting frequency ranges of functional groups and the literature frequency ranges presented in Table 2. Incorporating PXT results in modifying the symmetric area of C-H in STC EL, causing a minor shift from 2979 cm^− 1^ to 2974 cm^− 1^. The region of the symmetric C-H stretching vibration is determined by the number of gauche conformers in the hydrocarbon chains. This shift indicates an increase in the gauche conformers and alterations in the arrangement of the hydrocarbon chains [33, 73]. Any minor modifications in the symmetrical region can have significant effects because they are highly sensitive to any mobility or conformational changes in the hydrocarbon chain. Also, a diminishing intensity of the S = O peak was noticed after PX encapsulation. This might occur due to the expected interactions between the sulfur and the ester functional group of PX. A similar trend was noticed by incorporating PX with sulfur compounds [74]. For the empty T80 formulation, the C-O bonding of the ester group was seen at 1729 cm^− 1^. In addition, the C-O-C of the cyclic ether, usually noticed at 1140–1070 cm^− 1^, was represented at 1085 cm^− 1^. After PX encapsulation, the cyclic ether peak in the PX-loaded T80 EL diminished to a very small peak, and the ester peak faded. The reduction of the ether Peak and ester might have occurred due to the interaction between the PX and the edge activator; the PX structure comprises multiple proton donors (ex, N-H bond) that might lead to a breakage in the ether bond and convert them to O-H bond. The increase in the intensity of the O-H bond Peak after encapsulating PX can support this hypothesis.

**Table 2:** A comparison between the resulting frequency ranges of functional groups and the literature frequency ranges

Frequency range (cm^− 1^)	Functional group	Literature frequency range (cm^− 1^)	References
3218–3349 cm^− 1^	O–H stretching	3385 cm^− 1^	[37, 75]
2919 and 2850 cm^− 1^	CH2 symmetric stretching	2850 ± 1 cm^− 1^ and 2919 ± 1 cm^− 1^	[76]
1270 cm^− 1^	PO4 symmetric stretching vibrations	1250–1230 cm-^1^	[70, 76]
1643 cm^− 1^	NH bending	1600–1700 cm^− 1^	[77]
1085 and 1044 cm^− 1^	S = O asymmetric stretching	1350 and 950 cm^− 1^	[72]

#### Atomic force microscopy analysis

**Fig. 10:**
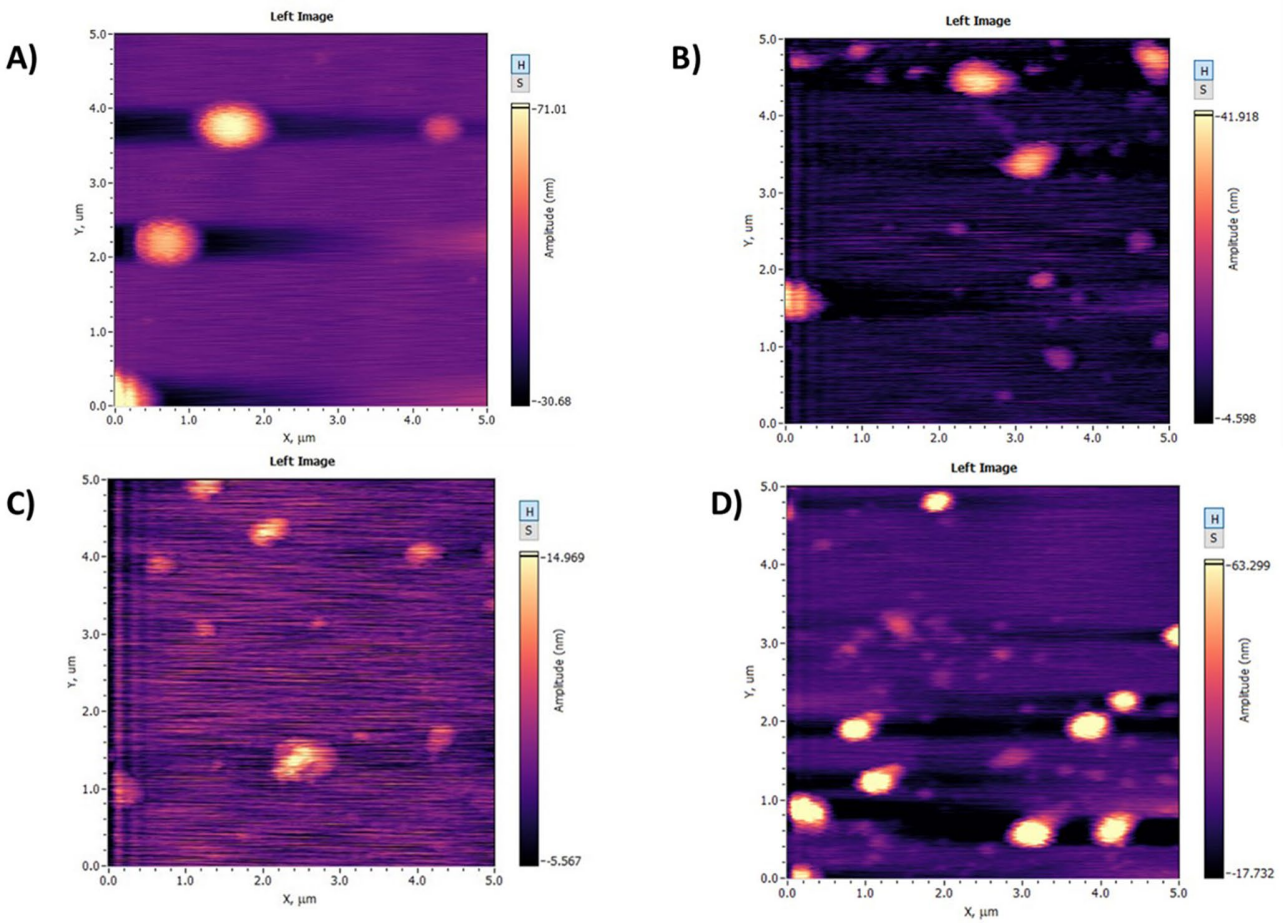
AFM images for (**A**) the empty T80 ELs, (**B**) the PX-loaded T80 ELs, (**C**) the empty STC ELs, (**D**) the PX-loaded STC ELs

The Atomic Forces microscope (AFM) is a valid technique to investigate the structural properties of the vesicles. AFM can provide a visual representation of the morphological structure of the liposomes. Several studies used the AFM as a well-established technique for nanometre imaging resolution [37, 78]. The current study uses the AFM to image the empty EL as standards and investigate any structural modifications after encapsulating PX. The images of empty STC EL showed a semi-circular and semi-organised shape compared to the Empty T80 EL, which represents a circular and organised structural shape (Fig. 10). After PX encapsulation, the shape of the STC vesicles becomes more circular and organised, contrary to the T80 vesicles, which become semi-circular and less organised after PX encapsulation. The AFM images of the T80 vesicles support the DLS results; an increase in PDI values of the T80 formulations was noticed after incorporating the PX. The less organised shape of the T80 formulations might be due to the packing of the PX between the bilayer of the liposomes, as the incorporation of PX within the bilayer may lead to a steric hindrance factor during the formation of the EL and create a bulk within the bilayer [67, 79].

#### Elasticity evaluation

The elasticity of the optimised EL formulations was tested to establish their deformability and flexibility. The proposed formulations are expected to demonstrate a higher degree of elasticity and flexibility due to the absence of cholesterol and the inclusion of various quantities of edge activators.

Conventional liposomes composed only of DOPC have been examined as a control to explore the effect of incorporating edge activators on the flexibility of the liposomes. By comparing the diameter of the ELs before and after the extrusion, the size of liposomes containing T80 or STC before and after pore passage isrelatively comparable (Table 3). These liposomes can efficiently cross the small pores, even if their size is approximately double the pore’s diameter. Contrary to conventional liposomes with 100% DOPC, conventional liposomes showed a reduced ability to pass through membranes with smaller pores than their own sizes, as they were withheld by the membrane. Thus, the DLS could not measure the liposomal formulation after extrusion. These findings are like previous studies investigating the variation in conventional and ELs elasticity [80, 81]. The disparity in deformability is likely attributed to the edge activator’s tendency towards highly curved structures, which consequently reduces the energy needed for particle deformation.

**Table 3:** Mean diameter (± S.D.) of different liposome formulations, before and after filtration through a microporous filter with pore diameter of 50 nm and liposomes membrane elasticity

Formulation	Lipid-edge activator ratio	Size before extrusion (nm)	Size after extrusion (nm)	Elasticity
Control	100% DOPC	140 ± 24	-	-
STC formulations	90:10	131 ± 12	100 ± 15	19
	70:30	127 ± 15	102 ± 9	20
T80 formulations	90:10	119 ± 17	107 ± 13	23
	70:30	115 ± 19	112 ± 11	25

Furthermore, a variation in the elasticity was exhibited with the different edge activators and the different utilised ratios (Table 3). There was a remarkable impact of the ratio of the edge activator to DOPC on elasticity; the higher elasticity was exhibited for the formulation with higher content of T80 and STC, which mimics the trend of several previous studies [6, 24, 64]. There is also a correlation between the type of edge activator used and the elasticity of the membrane. An investigation into the compositions of various edge activators showed that the formulations containing T80 exhibited more elasticity compared to the STC formulations. This might be attributed to the membrane insertion manner of each edge activator; the anionic edge activator STC gets into the membrane with planar steroidal moiety, aligning its planar steroidal part with the acyl chains of the phospholipids, while the hydrophilic side chain is directed towards the interface. The non-ionic edge activator T80 integrates into the membrane with its oleate residue (lipophilic part) in parallel with the acyl chains of the phospholipids. Meanwhile, the big head group of T80, which consists of around 20 polyoxymethylene units, faces the head group of the phospholipids [82]. The non-bulky and flexible hydrocarbon chain of the T80 could be the main reason for enhanced elasticity compared to the steroidal-like structure of the STC; since the encapsulated drug (PX)is highly hydrophobic, it will interact with the STC from the hydrophobic face, this kind of interaction may impede the mobility of STC in the membrane, resulting in a decrease in the elasticity of the liposomes [12]. However, Various parameters, including the composition, hydrocarbon chain of the lipid, kinds of edge activator, the polarity of the head group of the lipid and edge activator, the glass transition temperature of the lipid, and glycerol bridge as a link of acyl hydrocarbon influence the elasticity of lipid vesicles all collectively affect the fluidity and flexibility of the lipid bilayer in the vesicle system, which in turn enhances permeation through the microscopic pores of human skin [6].

#### Encapsulation efficiency

**Fig. 11:**
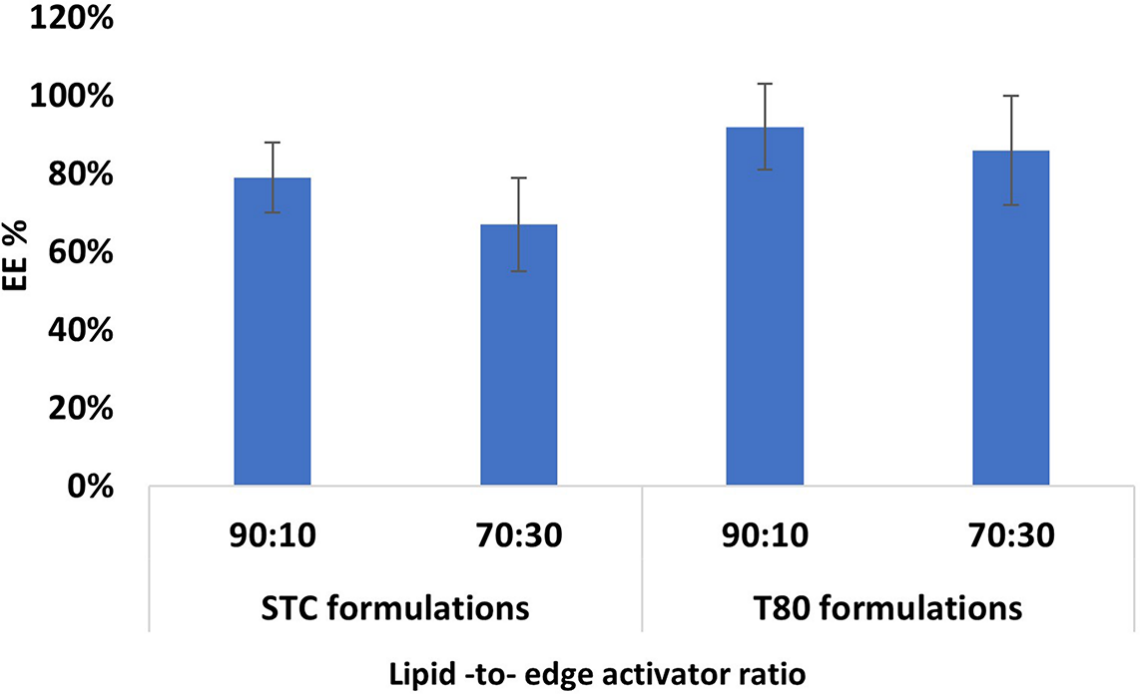
The EE% of the 90:10 and 70:30 lipid-to-edge activator of the T80 and STC formulations

The encapsulation efficiency (EE%) is crucial in determining the quantity of PX that can be encapsulated within a bilayer of elastic liposomes. The assessment of the EE % of PX within EL demonstrates the impact of employing various types and proportions of edge activators. The results indicate that the T80 formulations revealed a higher EE % of PX than the STC formulations (Fig. 11). The longer carbon chain of the T80 edge activator might be the main reason for the enhanced EE. As PX is a lipophilic molecule, it is hypothesised that increasing the carbon chain length of the edge activator increases the solubility of a lipophilic drug in the lipid bilayer, resulting in an increase in the EE [46, 83]. Several previous studies reported that increasing the carbon chain length enhanced the EE of lipophilic molecules [6, 13]. In a deeper look, the changing of the lipid-to-edge activator ratio significantly affects the EE% of PX. By comparing the 90:10 and 70:30 lipid-to-edge activator ratio, better EE% was reported with a 90:10 ratio in both T80 and STC formulations. The increase of the edge activator ratio from 90:10 to 70:30 showed a decline in the EE% (Fig. 10). This might be attributed to the high incidence of micelles formation with the 70:30; the micelles formation may lead to a decrease in the system’s overall EE due to micelles’ limited EE compared to the liposomes. [24, 46].

Generally, the edge activator type and ratio are not the only factors that can impact the EE%, as the manufacturing method has another significant impact. Since this work is the first in investigating the potential of MF systems in manufacturing the EL and assessing their EE%, the EE% of these ELs compared to the EE% results of EL manufactured using conventional methods, significant enhancement in the EE % of EL occurred by changing the manufacturing method form the conventional methods such as thin film hydration or vortexing-sonication to the microfluidic method. Al Shuwaili et al., have utilised the vortexing-sonication method to manufacture various PT-loaded ELs composed of lipids and different ratios of Tween or sodium cholate edge activators [24]. The maximum EE % between the various Tween formulations was 42%, and the average EE% of the diverse formulations was 31.4%. For the sodium cholate formulations, the maximum achieved EE% was 74%, and the average of the different formulations was 46%. Also, Utreja et al. employed the thin film hydration technique to manufacture PX-loaded EL composed of lipids and various ratios of span edge activators. The result showed that the average PX EE% within the EL was 53% [13].

#### In vitro release

**Fig. 12:**
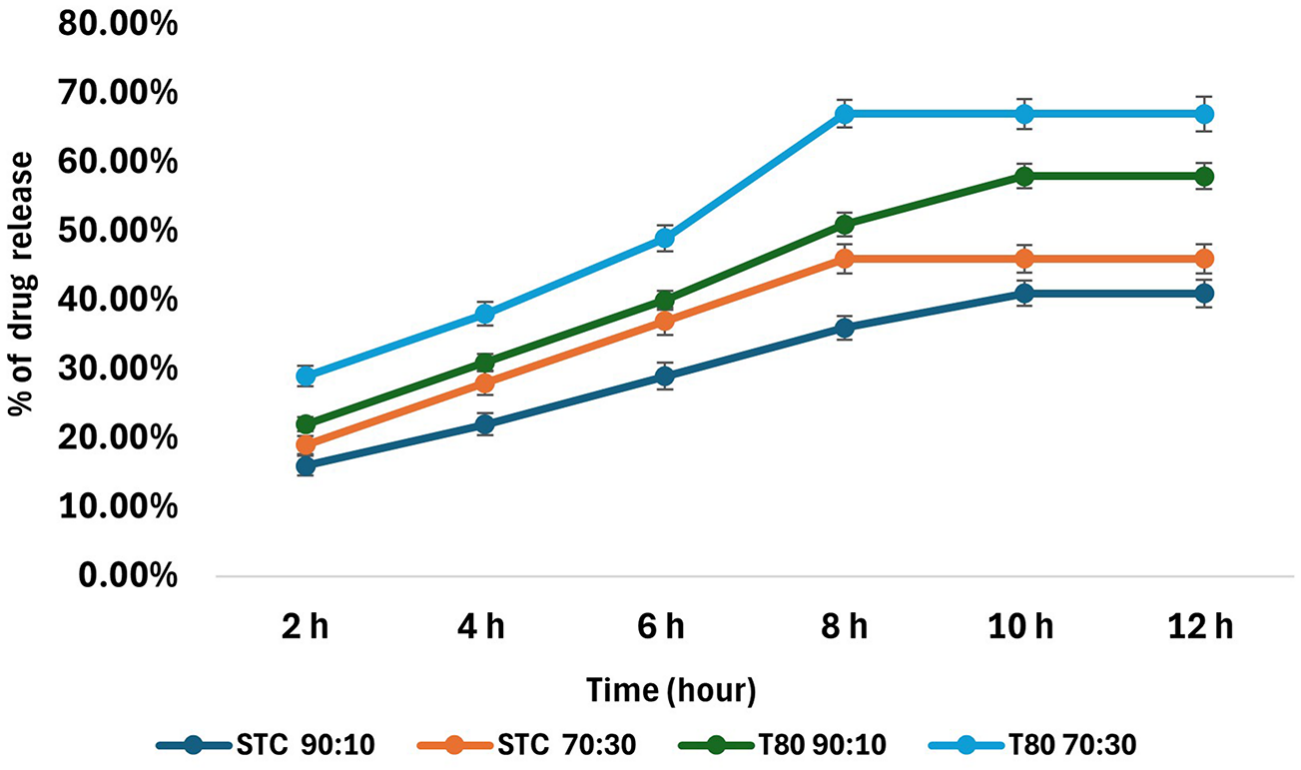
Drug release profile of the 90:10 and 70:30 lipid-to-edge activator ratio of the STC and T80 formulations

The release profile of PX from the STC and T80 formulations were tested for 12 h (Fig. 12). The results reveal an impact of the edge activator type and ratio on the release rate and the % of the released drug. Among both utilised edge activators, the T80 formulation achieved a higher % of released PX. The average % of released PX from the T80 formulation was 62.5% compared to the STC formulations with average 43.5%. This could be due to the more rigid vesicular structure of the STC ELs, which tightly entraps the medication and inhibits its release into the dissolution medium [24, 84]. Moreover, the higher EE of PX within the T80 formulation might be the essential reason for achieving the higher % of released PX. Multiple studies have emphasised the enhanced release of lipophilic drugs from T80 formulations, attributing this to the low rigidity and high permeability of the bilayer membrane [24, 85]. The various ratios of the utilised edge activator also impact the release rate and the % of released drugs. The results showed a slower drug release with low edge activator concentration; increasing the edge activator ratio from 90:10 to 70:30 increased the release rate. The 90:10 ratio of STC and T80 reached the steady state after 10 h compared to the 70:30 that reached the steady state after 8 h. The immediate release of the drug from formulations containing a higher concentration of the edge activator is attributed to the higher permeability of the bilayer membrane [24]. Generally, the T80 formulations were superior in drug release compared to other formulations, and optimum PX release was achieved by the 70:30 T80 formulation, which makes it a candidate for further studies.

## Conclusions

The experiments that were conducted highlight the positive impact of the MF system on enhancing the quality of the manufacturing of EL. The study investigates the effect of the TFR and FRR on the vesicle size, PDI, and stability. The increase of the FRR showed a significant decrease in the EL size and enhancement in the PDI values, while increasing TFR decreased the EL stability. The TFR 1 ml/min and 1:4 FRR were the optimum ratios for manufacturing empty EL. Also, Different types and concentrations of the T80 and STC edge activators have been investigated. Among the different investigated ratios, the 90:10 and 70:30 ratios were the optimum ratios for preparing EL. By changing the type of edge activator, T80 as neutral edge activator and STC as anionic edge activator showed a variation in size, PDI, ζ-potential, EE, and release. T80 formulations produced smaller size, neutral ζ-potential, higher EE (average 89%), and more immediate release compared to STC ELs. For the STC formulations, the average EE% was 79% and more anionic ζ-potential compared to T80 formulations was reported, which makes them suitable for transdermal applications. The formulations showed good stability for 4 weeks at various temperatures, including 4 °C, 25 °C, and 37 °C, especially after PX encapsulation. Further long-term stability studies can be performed, which might allow for a comprehensive understanding of EL behaviour and confirm the reliability of formulations. The outstanding characteristic of the optimised formulations showed a promising candidate for further development and In vivo studies. Therefore, this study’s findings are highly promising for the potential use of PX-loaded EL for transdermal applications for several types of skin cancer, including melanoma and carcinoma. Several additional assessments can be carried out to strengthen and reinforce the optimum EL characterization. Using the CryoTEM microscope can provide a more precise characterization of EL by visualising its interior structures. This method also allows for obtaining higher-resolution images, which can aid in identifying changes in vesicle size, shape, and distribution among different formulations.

## Electronic supplementary material

Below is the link to the electronic supplementary material.

Supplementary Material 1

## Data Availability

The datasets generated during and/or analysed during the current study are available from the corresponding author on reasonable request.
