# Supplementary material for: Paclitaxel-loaded elastic liposomes synthesised by microfluidics technique for enhance transdermal delivery
Source: Drug Deliv Transl Res. 2024 Jul 17;15(4):1265–83. doi: 10.1007/s13346-024-01672-0 (PMC11870984; doi:10.1007/s13346-024-01672-0)
Supplement: Supplementary file 1 — Supplementary Material 1 [file 13346_2024_1672_MOESM1_ESM.docx]

**Supplementary information**

**Title: Paclitaxel-loaded Elastic liposomes synthesised by microfluidics technique for enhance transdermal delivery**

Eman Jaradat^1^, Adam Meziane^2^, Dimitrios A. Lamprou^1^*

^1^School of Pharmacy, Queen’s University Belfast, 97 Lisburn Road, Belfast BT9 7BL, UK

^2^Fluigent, 94270 Le Kremlin-Bicêtre, France

* Correspondence: [d.lamprou@qub.ac.uk](mailto:d.lamprou@qub.ac.uk)

Table S1: Composition and Microfluidic parameters of the various elastic formulations.

| **Formulation code** | **Composition** | **TFR** | **FRR** |
| --- | --- | --- | --- |
| F1-STC | DOPC : STC  90:10 | 1 ml/min | 1:2 |
| F2- STC |  |  | 1:3 |
| F3-STC |  |  | 1:4 |
| F4-STC |  | 2 ml/min | 1:2 |
| F5-STC |  |  | 1:3 |
| F6-STC |  |  | 1:4 |
| F7-STC | DOPC :STC  70:30 | 1 ml/min | 1:2 |
| F8 -STC |  |  | 1:3 |
| F9-STC |  |  | 1:4 |
| F10-STC |  | 2 ml/ min | 1:2 |
| F11- STC |  |  | 1:3 |
| F12 -STC |  |  | 1:4 |
| F13-STC | DOPC :STC  50:50 | 1 ml/min | 1:2 |
| F14-STC |  |  | 1:3 |
| F15-STC |  |  | 1:4 |
| F16-STC |  | 2 ml /min | 1:2 |
| F17-STC |  |  | 1:3 |
| F18-STC |  |  | 1:4 |
| F1 -T80 | DOPC : T80  90:10 | 1 ml/min | 1:2 |
| F2-T80 |  |  | 1:3 |
| F3-T80 |  |  | 1:4 |
| F4-T80 |  | 2 ml/min | 1:2 |
| F5-T80 |  |  | 1:3 |
| F6-T80 |  |  | 1:4 |
| F7-T80 | DOPC : T80  70:30 | 1 ml/min | 1:2 |
| F8-T80 |  |  | 1:3 |
| F9-T80 |  |  | 1:4 |
| F10-T80 |  | 2 ml/min | 1:2 |
| F11-T80 |  |  | 1:3 |
| F12-T80 |  |  | 1:4 |
| F13-T80 | DOPC : T80  50:50 | 1 ml/min | 1:2 |
| F14-T80 |  |  | 1:3 |
| F15-T80 |  |  | 1:4 |
| F16-T80 |  | 2 ml/min | 1:2 |
| F17-T80 |  |  | 1:3 |
| F18-T80 |  |  | 1:4 |

Figure S1: Zeta-potential of empty Sodium taurocholate hydrate elastic liposome formulations.

Figure S2: Zeta-potential of Tween 80 elastic liposomes at FRR 1:2,1;3, and 1:4.

Figure S3: Stability studies over 4-weeks period of 90:10 sodium taurocholate elastic liposomes at TFR 1 ml/min and FRR 1:2.

Figure S4: Stability studies over 4-weks period of 90:10 sodium taurocholate hydrate elastic liposomes at TFR 1 ml/min and FRR 1:3.

Figure S5: Stability studies over 4-weeks period of 90:10 sodium taurocholate hydrate elastic liposomes at TFR 1 ml/min and FRR 1:4.

Figure S6: Stability studies over 4-weeks period of 70:30 sodium taurocholate hydrate elastic liposomes at TFR 1 ml/min and FRR 1:2.

Figure S7: Stability studies over 4-weeks period of 70:30 sodium taurocholate hydrate elastic liposomes at TFR 1 ml/min and FRR 1:3.

Figure S8: Stability studies over 4-weeks period of 70:30 sodium taurocholate hydrate elastic liposomes at TFR 1 ml/min and FRR 1:4.

Figure S9: Stability studies over 4-weeks period of 90:10 Tween 80 elastic liposomes at TFR 1 ml/min and FRR 1:2.

Figure S10: Stability studies over 4-weeks period of 90:10 Tween 80 elastic liposomes at TFR 1 ml/min and FRR 1:3.

Figure S11: Stability studies over 4-weeks period of 90:10 Tween 80 elastic liposomes at TFR 1 ml/min and FRR 1:4.

Figure S12: Stability studies over 4-weeks period of 70:30 Tween 80 elastic liposomes at TFR 1 ml/min and FRR 1:3.

Figure S13: Stability studies over 4-weeks period of 70:30 Tween 80 elastic liposomes at TFR 1 ml/min and FRR 1:4.

Figure S14: Stability studies over 4-weeks period of PX-loaded sodium taurocholate hydrate elastic liposomes at TFR 1 ml/min and FRR 1:4.

Figure S15: Stability studies over 4-weeks period of Paclitaxel-loaded Tween 80 elastic liposomes at TFR 1 ml/min and FRR 1:4.


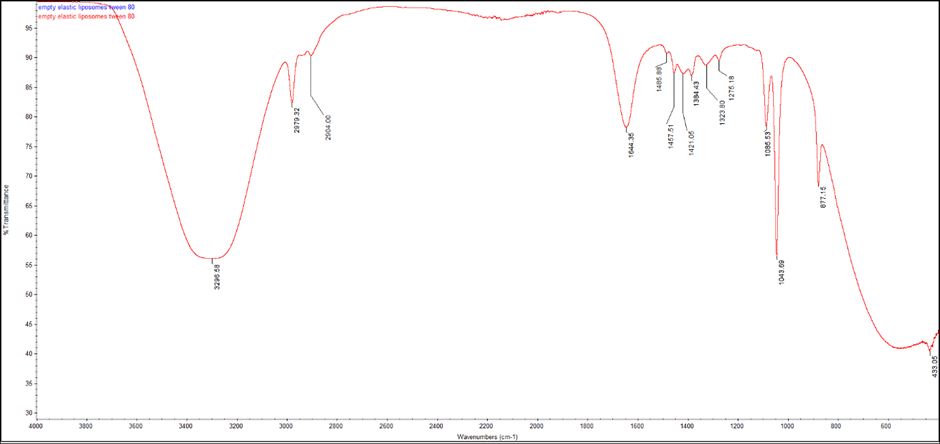


Figure S16: FTIR spectra of empty sodium taurocholate hydrate elastic liposomes.


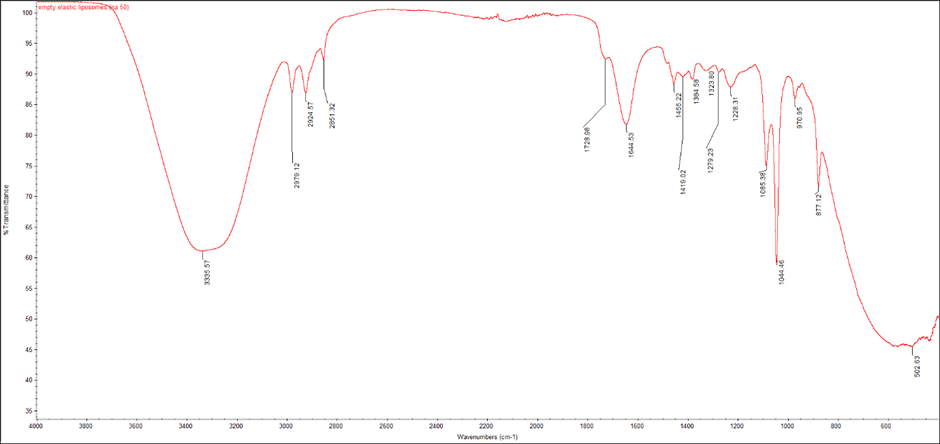


Figure S17: FTIR spectra of the empty Tween 80 elastic liposomes.


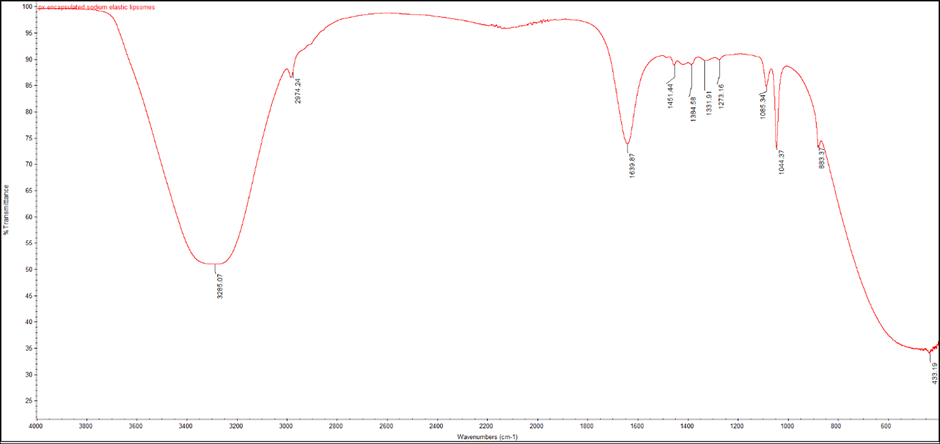


Figure S18 : FTIR spectra PX-loaded sodium taurocholate hydrate elastic liposomes.


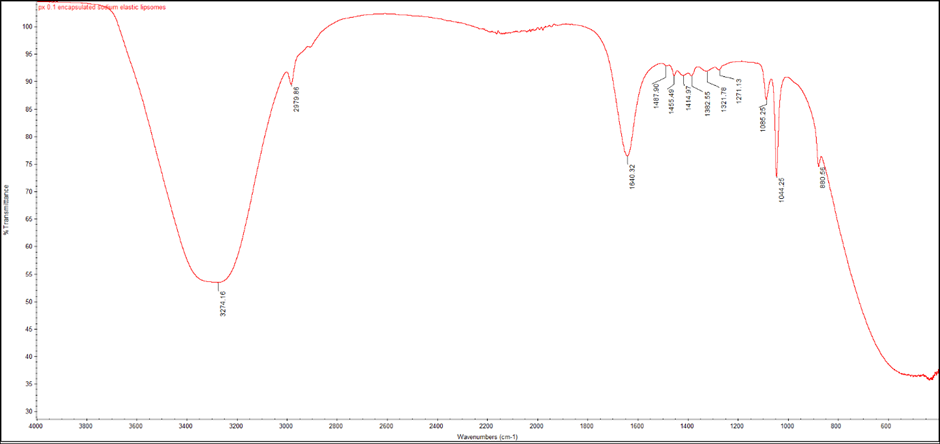


Figure S19 : FTIR spectra PX-loaded Tween 80 elastic liposomes.
